# Design, Synthesis and Structural Analysis of Glucocerebrosidase Imaging Agents

**DOI:** 10.1002/chem.202102359

**Published:** 2021-10-29

**Authors:** Rhianna J. Rowland, Yurong Chen, Imogen Breen, Liang Wu, Wendy A. Offen, Thomas J. Beenakker, Qin Su, Adrianus M. C. H. van den Nieuwendijk, Johannes M. F. G. Aerts, Marta Artola, Herman S. Overkleeft, Gideon J. Davies

**Affiliations:** ^1^ Department of Chemistry, York Structural Biology Laboratory (YSBL) University of York Heslington York YO10 5DD UK; ^2^ Department of Bio-organic Synthesis, Leiden Institute of Chemistry Leiden University Einsteinwegg 55 2300 RA Leiden Netherlands; ^3^ Department of Medicinal Biochemistry, Leiden Institute of Chemistry Leiden University Einsteinwegg 55 2300 RA Leiden Netherlands

**Keywords:** Activity-based probes, Carbohydrates, Cyclitols, Fluorescent Probes, β-Glucocerebrosidase, Inhibitors, Structural Biology

## Abstract

Gaucher disease (GD) is a lysosomal storage disorder caused by inherited deficiencies in β‐glucocerebrosidase (GBA). Current treatments require rapid disease diagnosis and a means of monitoring therapeutic efficacy, both of which may be supported by the use of GBA‐targeting activity‐based probes (ABPs). Here, we report the synthesis and structural analysis of a range of cyclophellitol epoxide and aziridine inhibitors and ABPs for GBA. We demonstrate their covalent mechanism‐based mode of action and uncover binding of the new *N‐*functionalised aziridines to the ligand binding cleft. These inhibitors became scaffolds for the development of ABPs; the O6‐fluorescent tags of which bind in an allosteric site at the dimer interface. Considering GBA's preference for O6‐ and *N*‐functionalised reagents, a bi‐functional aziridine ABP was synthesized as a potentially more powerful imaging agent. Whilst this ABP binds to two unique active site clefts of GBA, no further benefit in potency was achieved over our first generation ABPs. Nevertheless, such ABPs should serve useful in the study of GBA in relation to GD and inform the design of future probes.

## Graphical abstract

Tagged cyclophellitols offer a powerful activity‐based probe (ABP) approach to the study of glycosidase activities. Here, a range of cyclophellitol epoxide and aziridine inhibitors and ABPs were synthesised and investigated on the 3D structure of human β‐glucocerebrosidase (GBA), demonstrating their mechanism‐based mode of action. We envisage these inactivators will serve useful in the study of GBA in relation to Gaucher Disease.

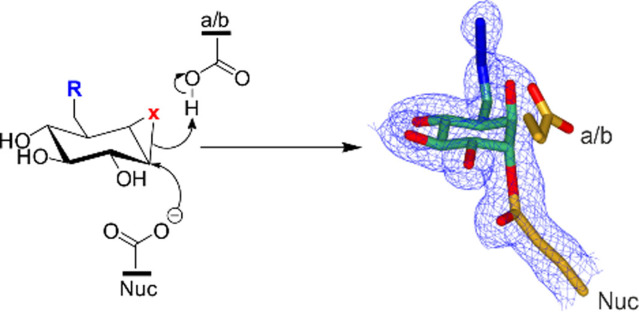

## Introduction

Gaucher disease (GD) is the most common lysosomal storage disorder which is caused by inherited deficiencies in β‐glucocerebrosidase (glucosylceramidase, GCase, GBA, EC 3.2.1.45). This lysosomal glycoside hydrolase is encoded by the *GBA1* gene^[1]^ and according to The Human Gene Mutation Database (www.hgmd.org, Institute of Medical Genetics in Cardiff^[2]^) over 500 genetic mutations at the *GBA1* locus are known. Moreover, mutations in the *GBA1* gene have recently been identified as the highest known genetic risk factor for Parkinson's disease (PD),[^3^, ^4^, ^5^, ^6^] further intensifying therapeutic interest in the *GBA1* gene and enzyme.

GD is primarily characterized by the cellular accumulation of glucosylceramide (GlcCer), and its deacylated derivative gluco‐sylsphingosine (GlcSph), as a result of deficient GBA activity.[^7^, ^8^, ^9^] The multisystemic storage of these glycolipids leads to the clinical symptoms of GD, which can vary considerably in frequency and severity. Clinical manifestation of GD type 1 and GD type 2 commonly include skeletal disease and visceral disease affecting the spleen, kidneys, liver and heart.[^10^, ^11^, ^12^, ^13^] In more severe cases (GD type 3), neurological disorders also arise due to GlcCer deposition in the brain.[^14^, ^15^]

GBA is a 497 amino‐acid membrane‐associated glycoprotein belonging to GH30 CAZy family (www.cazy.org) of retaining β‐glucosidases.^[16]^ GBA is primarily responsible for catalyzing the degradation of GlcCer by hydrolytic cleavage of the β‐glucose moiety from the aglycone to yield free ceramide and glucose.[^7^, ^17^, ^18^] This is achieved with retention of β‐anomeric stereochemistry of the released glucose unit through a Koshland double displacement mechanism using Glu340 as the catalytic nucleophile and Glu235 as the catalytic acid‐base. The enzymatic nucleophile of GBA was identified (and corrected from Asp443^[19]^) by the Wither's lab through covalent‐trapping of the enzyme with a 2‐fluoro‐2‐deoxy glucoside inactivator.^[20]^ Given the clinical importance of GBA in both GD and PD, it is arguably the most widely studied human glucosidase, with relentless interest in developing novel chaperones,[^21^, ^22^, ^23^, ^24^] inhibitors[^25^, ^26^, ^27^] and activity‐based probes (ABPs)[^28^, ^29^] to study this enzyme in disease pathogenesis, diagnosis and treatment.

The seminal 3D structure of GBA was reported in 2003,^[30]^ followed by a number of co‐crystal complexes with imino‐sugar inhibitors *N*‐butyldeoxynojirimycin and *N*‐nonyldeoxy‐nojirimycin.^[31]^ Later studies have investigated GBA at the 3D level to obtain insight into GD mutations and potential molecular chaperone binding motifs.[^32^, ^33^, ^34^, ^35^] For example, active site directed quinazoline modulators,[^21^, ^36^] competitive 3,4,5,6‐tetra‐hydroxyazepane inhibitors^[37]^ and uncompetitive pyrrolo[2,3‐b]pyrazines inhibitors^[38]^ have recently been reported.

**Figure 1:**
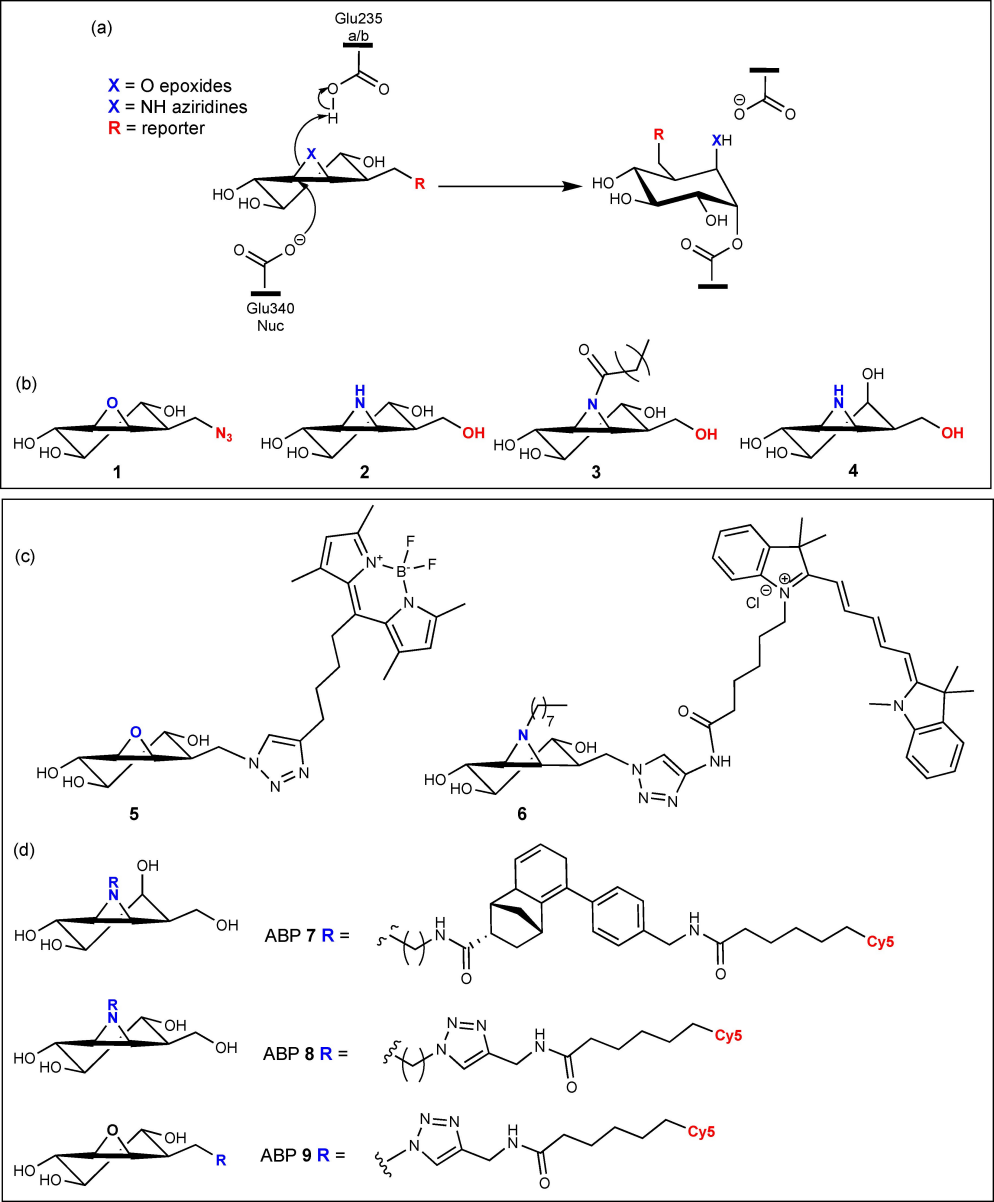
(a) Reaction mechanism of GBA inactivation by irreversible cyclophellitol epoxide and aziridine inhibitors. Panel of (b) cyclophellitol epoxide and aziridine inhibitors (**1**–**4**) and (c) activity‐based probes (ABPs) (**5**–**6**) structurally investigated in this work. (d) Range of *galacto‐* and *gluco‐*configured cyclophellitol ABPs (**7**–**9**) employed for comparative ABPP studies in this work.

Cyclophellitol, a natural product originally isolated from the mushroom *Phellinus sp*.,^[39]^ is a potent and irreversible β‐glucosidase inhibitor which exhibits considerably improved selectivity over its close structural homologue conduritol‐B‐epoxide (CBE[^40^, ^41^]).^[42]^ Building on this enhanced selectivity, and with inspiration from the Withers’ fluoro‐glycosides,[^43^, ^44^, ^45^] we have developed a range of cyclophellitol‐based inhibitors and ABPs for GBA (Figure 1a,b). Indeed, tagged‐cyclophellitol epoxides and aziridines provide a powerful activity‐based protein profiling (ABPP) approach to the study of GBA both *in situ* and *in vitro*,[^28^, ^46^] with potential applications in diagnostics and therapeutic evaluation. In a previous study, we touched upon the binding of a O6‐adamantane substituted cyclophellitol inhibitor and a Cy5‐tagged cyclophellitol ABP for GBA,^[27]^ however, these structures presented just a fraction of our structural work on the cyclophellitol‐based inactivators. In fact, most of these inhibitors and their recent ABP iterations have not been observed on the 3D structure of GBA, hindering a fundamental understanding of ABP reactivity, specificity and conformation.

Here, we sought to structurally dissect the binding of various cyclophellitol‐based inhibitors and ABPs with human GBA, comparing epoxides vs. aziridines (**1**, **5** vs. **2**, **3**, **4**, **6**), *gluco*‐ vs. *galacto*‐configuration (**1**, **2**, **3**, **5**, **6** vs. **4**, **7**), aziridine nitrogen functionalization (**3**, **6**), O6‐functionalisation with fluorescent reporter groups (BODIPY **5** and Cy5 **6**) and bi‐functionalization at both the O6‐position and aziridine nitrogen (**6**), (Figure 1b,c). In doing so, we hoped to establish the binding mode of these inactivators and uncover key mechanistic and structural information to inform the design of more efficacious probes for studying human GBA with regards to GD.

## Results and Discussion

Tagged cyclophellitol epoxides and aziridines are valuable tools for studying the activity of retaining glycosidases in a wide range of applications, from biomedical purposes in human health and disease[^27^, ^47^, ^48^] to biotechnology for the study of biomass degrading enzymes.[^49^, ^50^, ^51^] Here, we report the design, synthesis and structural analysis of a panel of β‐glucose configured cyclophellitol‐based inhibitors and ABPs on the 3D structure of GBA with supportive ABPP studies (Figure 1).

### Unsubstituted cyclophellitol epoxide (1)

**Figure 2:**
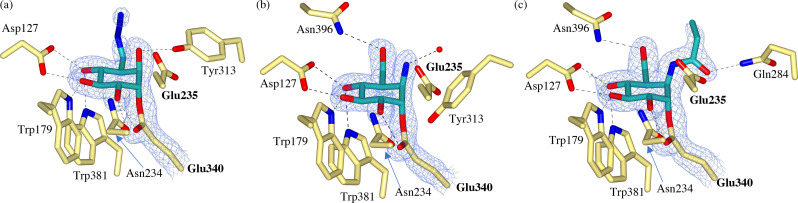
Observed electron density for **1**, **2** and **3** bound covalently to the catalytic nucleophile (Glu340) of rhGBA through trans‐diaxial ring opening of the epoxide or aziridine warhead. Weak electron density observed for the C6‐azide tag of **1** but clear density for ring‐opened *N*‐acyl aziridine of **3**. Maximum‐likelihood/σA weighted (2F_o_–F_c_) electron density maps contoured to 1.2 σ (a=1.30 e^−^/Å^3^, b=1.38 e^−^/Å^3^, c=1.30 e^−^/Å^3^).

A co‐crystal structure of recombinant human GBA (rhGBA) in complex with azide‐tagged cyclophellitol **1** was obtained at 1.70 Å resolution, revealing classical trans‐diaxial ring‐opening of the epoxide warhead to form a covalent enzyme‐inhibitor complex with the enzymatic nucleophile (Glu340), (Figure 2a). The covalent bond length was measured to be 1.42 Å, with the reacted cyclitol adopting the ^4^C_1_ chair conformation. This enzyme‐inhibitor complex is consistent with the β‐glucoside conformational reaction itinerary (which follows a Michaelis Complex→Transition state^≠^→Covalent Intermediate itinerary of ^1^S_3_→^4^H_3_→^4^C_1_) and is consistent with the revised conduritol‐β‐epoxide (CBE) complex,^[52]^ which was corrected from the originally reported boat conformation.^[53]^ Additionally, the cyclophellitol moiety forms an extensive hydrogen bonding network within the active site, making hydrogen bonds with Asp127, Trp179, Asn234, Tyr313 and Trp381. Whilst electron density for the C6 azido‐tag was weak, likely due to flexibility and disorder, there was sufficient density to model the azide substituent extending ‘upwards’ into a relatively spacious cavity at the back of the active site. An absence of azide electron density for inhibitor **1** has been reported previously when in complex with an unrelated bacterial β‐glucoside (*Thermotoga maritima*, T*m*GH1).^[54]^ In contrast to this bacterial co‐complex, the improved azido‐electron density of **1** in complex with rhGBA reported here provides some insight into the conformation of the azide‐tag, which may serve as a ligation handle for two step activity‐based protein profiling (ABPP).[^55^, ^56^] Indeed, further functionalization of this azide‐tag for two‐step labelling is structurally supported by the relatively large open cavity in which the azide‐substituent binds, which would likely accommodate larger reporter groups. However, in the case of GBA, direct one‐step ABPs have proved more effective than two‐step strategies for activity‐based profiling.^[56]^

### Unsubstituted cyclophellitol aziridine (2)

Configurationally isomeric cyclophellitol epoxides and aziridines are often considered interchangeable, although not always equally potent,[^29^, ^57^] as inhibitors of retaining glycosidases. Therefore, to probe whether this holds true from a mechanistic inhibition point of view, a crystal structure rhGBA in complex with cyclophellitol aziridine **2** was obtained at 1.70 Å resolution to enable a detailed comparison with cyclophellitol epoxide **1**.

The electron density of the resulting co‐crystal structure unambiguously shows that **2** reacts with the enzymatic nucleophile of GBA (Glu340) through the aziridine warhead to form a covalent trans‐diaxial ring opened complex which is essentially identical to reacted cyclophellitol epoxide **1**, (Figure 2b). Indeed, **2** forms a covalent complex in the ^4^C_1_ chair conformation with a covalent bond length of 1.46 Å to Glu340. The only notable difference between the complex of **2** and **1** lies in their hydrogen bonding networks, where a hydrogen bond is formed between the O6‐hydroxyl of **2** and Asn396, and the hydrogen bond to Tyr313 is lost, likely due to the “downwards” displacement of Tyr313 in complex with **2**. The flexibility of Tyr313 has been reported previously^[58]^ and the “downwards” conformation observed in complex with **2** appears to preclude hydrogen bonding with the ring‐opened aziridine. Instead, the aziridine warhead forms a hydrogen bond to a nearby water molecule in the active site. Overall, this co‐crystal structure demonstrates the almost identical binding mode of cyclophellitol epoxides and aziridines, which supports their interchangeable use in the development of ABPs for GBA. Indeed, both fluorescently‐tagged cyclophellitol epoxides and aziridines have proved effective, nanomolar probes for GBA (apparent IC_50_ of 1.24 nM for BODIPY‐functionalized epoxide compared to IC_50_ of 1.15 nM for BODIPY‐functionalized aziridine^[29]^), with great potential for the study of GBA in regards to GD.

### *N*‐Acyl cyclophellitol aziridine (3)

One route to modifying cyclophellitol aziridines and facilitating their conversion to ABPs is functionalization of the aziridine nitrogen.^[59]^ Consequently, *N*‐acyl functionalized aziridine **3** was synthesized and found to be a more potent inhibitor than the unsubstituted aziridine analogue **2** (apparent IC_50_ of 0.07 μM for *N*‐acyl **3** compared to the 0.5 μM for unsubstituted **2** ^[57]^). In light of this improved potency, we sought to investigate the structural accommodation of the *N‐*acyl functionalization by GBA.

A co‐crystal structure of rhGBA in complex with **3** was obtained at 1.76 Å resolution to reveal unambiguous electron density for the reacted cyclitol bound covalently to the enzymatic nucleophile via trans‐diaxial ring opening of the *N*‐acyl aziridine warhead, (Figure 2c). The cyclophellitol ring adopts the expected ^4^C_1_ chair conformation in the resulting complex, with a covalent bond length of 1.42 Å to Glu340. This complex is superimposable with that formed by the unsubstituted aziridine analogue **2**. Importantly, the electron density for the *N*‐acyl group and subsequent 2–3 carbon tail was sufficient to model binding of the ring opened *N*‐acyl chain to a narrow active site cleft flanked by Tyr313 and Glu284. The carbonyl oxygen of the *N‐*acyl aziridine also forms a hydrogen bond with Gln284, introducing an additional hydrogen bond to the cyclophellitol aziridine network. It's possible that binding of this *N*‐acyl moiety somewhat mimics the binding of one of the two acyl chains of the natural GlcCer substrate, which may provide a structural basis for the improved potency reported for *N‐*acyl aziridines over the unsubstituted aziridine analogue.[^57^, ^59^] Furthermore, *N‐*alkyl functionalized aziridines have also proved to be potent and selective inhibitors of GBA (apparent IC_50_ of 0.017 μM for *N*‐alkyl compared to IC_50_ of 0.07 μM for *N*‐acyl^[57]^), with improved chemical stability over their *N*‐acyl equivalents.^[59]^ This highlights the potential for future inhibitor and ABP development through *N*‐acylation/alkylation of cyclophellitol aziridines.

### Cross‐reactivity with *galacto‐*configured aziridines

One issue surrounding ABPs is their occasional cross‐reactivity with related glycosidases. Of note, GBA can be by inhibited both *gluco*‐ and *xylo*‐configured substrates, with demonstrated activity against 4‐methylumbelliferyl‐β‐xyloside;^[60]^ conversely, *galacto*‐configured cyclophellitol epoxides have proved inactive against GBA.^[61]^ Nevertheless, studies on other glycosidases have shown that *galacto*‐configured inactivators occasionally bind to glucosidases. For example, Gloster et al., 2007 reported binding of T*m*GH1 β‐glucoside to *galacto*‐hydroximolactam.^[62]^ Therefore, whilst we know *galacto‐*configured cyclophellitol epoxides do not bind GBA, we sought to assess if there is potential for cross‐reactivity with *galacto*‐configured aziridine inhibitor **4** and ABP **7**.

### Synthesis of Cy5 Tagged *Galacto*‐Configured Cyclophellitol Aziridine (7)

**Scheme 1:**
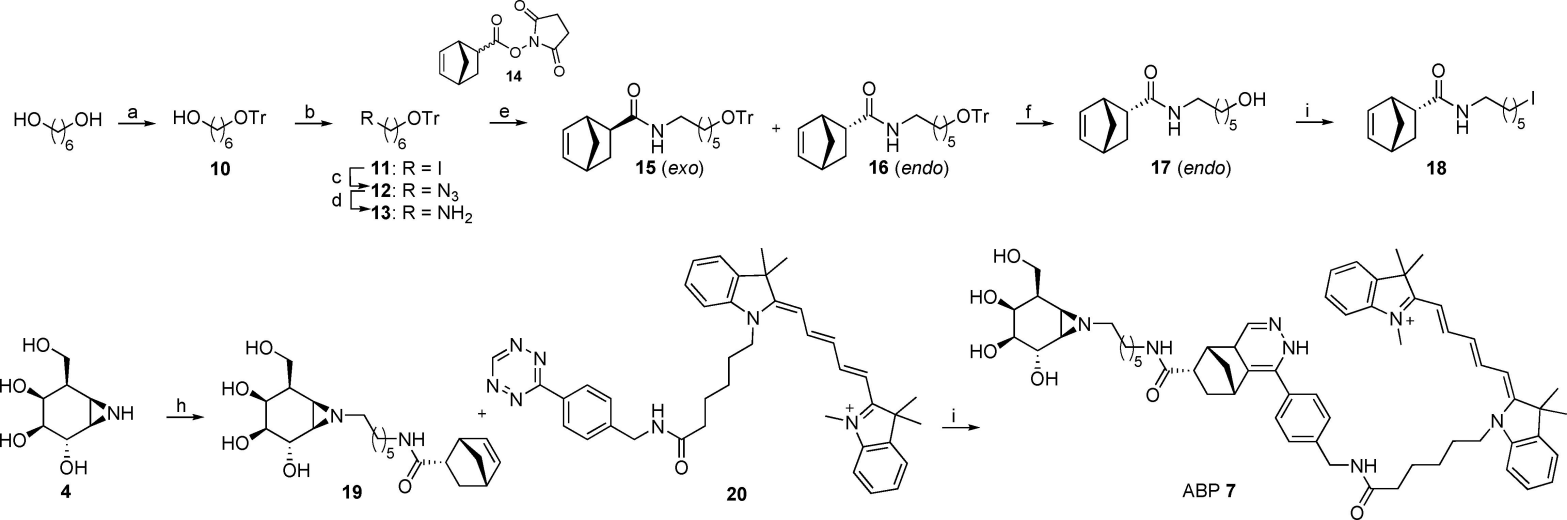
Synthesis of ABP **7**. Reagents and conditions: a) TrCl, pyridine, CH_2_Cl_2_, rt, 90 min, 95 %; b) imidazole, PPh_3_, I_2_, Et_2_O, CH_3_CN, rt, overnight, 80 %; c) NaN_3_, DMF, 80 °C, overnight, quant; d) polymer‐bound PPh_3_, H_2_O, THF, 48 h, quant; e) norbornene‐OSu, DIPEA, DCE, rt, overnight, **15**: 28 %, **16**: 68 %; f) *p*‐toluenesulfonic acid, CH_2_Cl_2_, MeOH, rt, overnight, 86 %; g) PPh_3_, I_2_, imidazole, THF, reflux, 1.5 h, 73 %; h) **18**, K_2_CO_3_, DMF, 75 °C, overnight, 12 %; i) Cy5 tetrazine **20**, MeOH, overnight, 87 %.

β‐Galactose‐configured cyclophellitol aziridine **4**, prepared as described previously,^[63]^ was alkylated with 8‐azido‐1‐iodooctane under basic conditions (Scheme 1). Unfortunately, Cu(I)‐catalyzed azide/alkyne cycloaddition (CuAAC) with Cy5‐alkyne proved abortive and a complex mixture was obtained. In an attempt to obtain a β‐galactosidase ABP, the inverse‐electron‐demand Diels‐Alder (IEDDA) ligation strategy was investigated to introduce the fluorophore at the final synthetic step. For this purpose, norbornene‐modified cyclophellitol aziridine **19** was synthesized and reacted with tetrazine‐Cy5 **20** to obtain ABP **7**. The synthesis of norbornene‐functionalized aziridine started with monotritylation of 1,6‐hexanediol to give **10** followed by iodination of the primary alcohol with iodine and substitution with sodium azide to afford **12**. Reduction of azide **12** using triphenylphosphine on beads gave amine **13**. Norbornene‐OSu ester **14** was obtained according to the literature procedure^[64]^ as a mixture of *endo‐* and *exo‐*isomers. This mixture was condensed with amine **13** resulting in a mixture of norbornene‐trityl products (*exo/endo*, 1 : 2.3), which were easily separated by silica gel column chromatography. Deprotection of *endo‐*product **16** under acidic conditions gave alcohol **17** in 86 % yield. Treatment of **17** with triphenylphosphine, iodine and imidazole at elevated temperatures in tetrahydrofuran. afforded iodonorbornene **18**. Cyclophellitol aziridine **7** was then *N*‐alkylated with iodonorbornene linker **18** using potassium carbonate as base in DMF. Subsequent ligation^[65]^ with Cy5 tetrazine **19** gave ABP **7** as mixture of isomers.

### In‐solution labelling of rhGBA by *galacto‐*configured cyclophellitol aziridine ABP 7

**Figure 3:**
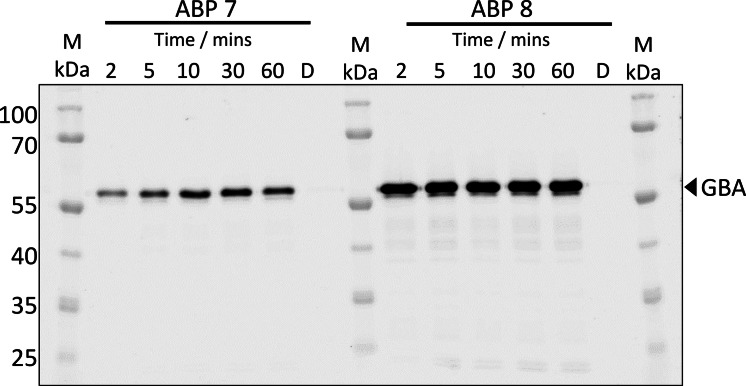
Time dependent labelling of rhGBA (700 nM) by Cy5‐tagged *galacto‐*ABP **7** (150 nM) after 2, 5, 10, 30 and 60 mins showing that labelling reaches saturation at 30 mins. In comparison, labelling by Cy5‐tagged *gluco*‐ABP **8** (150 nM) reaches saturation in 2mins. D=denatured protein sample demonstrating there is no unspecific labelling with inactive rhGBA. ABP labelled rhGBA (60 kDa) visualized by Cy5 fluorescent readout.

To rapidly investigate for cross‐reactivity with *galacto*‐configured cyclophellitol aziridines, time‐dependent labelling assays of rhGBA with *galacto‐*configured Cy5‐tagged aziridine ABP **7** were performed (Figure 3).

To our surprise, ABP **7** (150 nM) rapidly labelled rhGBA within 2 minutes and reached saturation within 30‐minutes (Figure 3). In comparison, labelling by *gluco‐*configured ABP **8** (150 nM) achieved saturation within 2 minutes (Figure 3). Whilst *galacto‐*configured ABP **7** is slower to label rhGBA compared to *gluco‐*configured ABP **8**, this simple labelling assay demonstrates the cross‐reactivity potential of β‐*galacto*‐configured aziridine ABPs with GBA and suggests that such ABPs should be carefully analysed for cross reactivity before interpretation *in vivo*. However, it should also be noted that broad spectrum ABPs have proved useful in enzyme and inhibitor discovery, illustrating that not all ABPs need to be highly specific.^[66]^ Nevertheless, it is important to establish the reactivity and binding of such ABPs to understand their limitations and identify areas for future improvements. Therefore, cross‐reactivity data such as this are important in guiding the selection of ABPs for a desired application.

### *Galacto‐*configured cyclophellitol aziridine (4)

**Figure 4:**
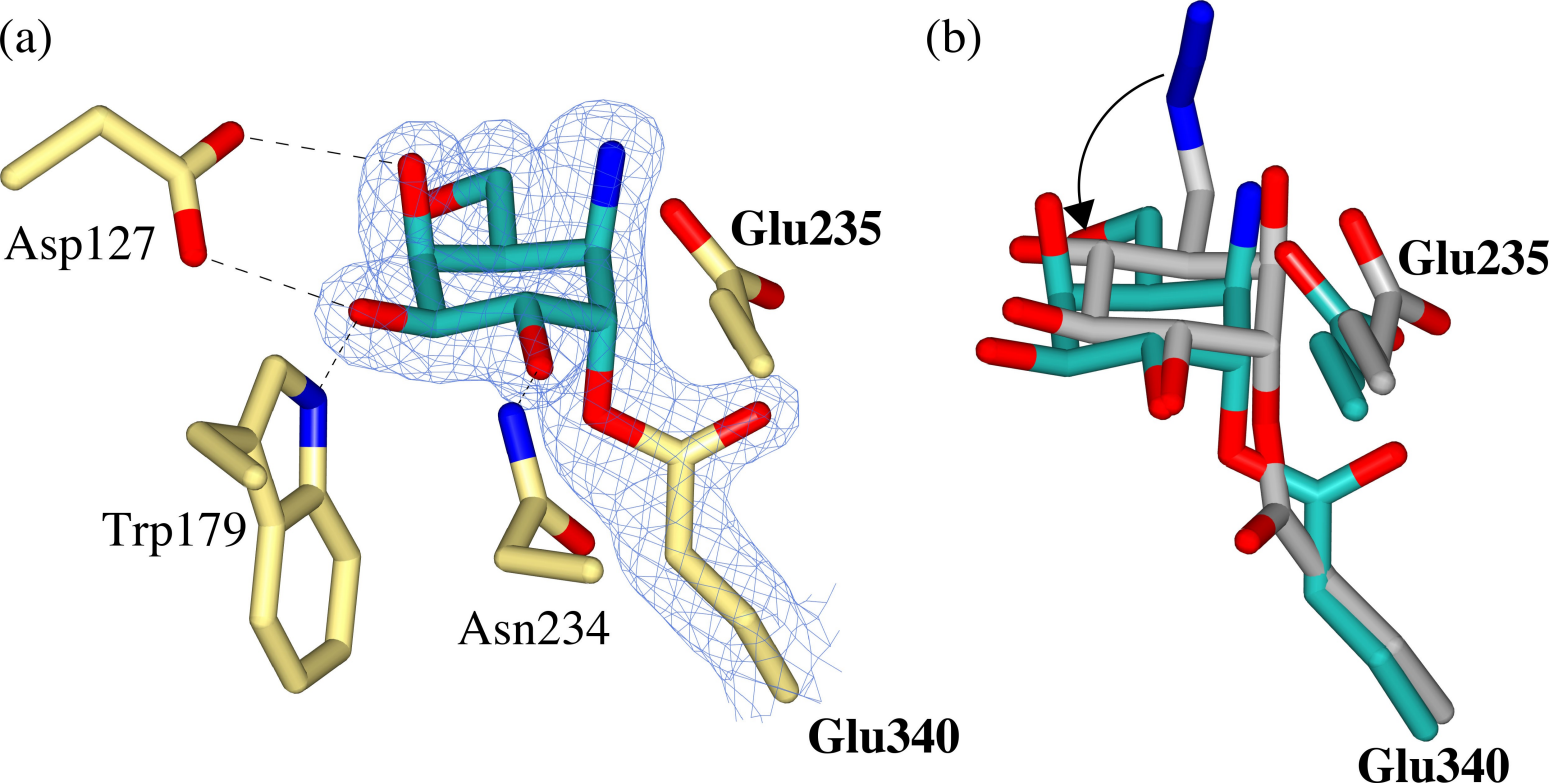
(a) Observed electron density for *galacto‐*configured aziridine **4** bound covalently to the catalytic nucleophile (Glu340) of rhGBA through trans‐diaxial ring opening of the aziridine warhead. Maximum‐likelihood/σA weighted (2F_o_–F_c_) electron density map contoured to 1.2 σ (1.31 e^−^/Å^3^) (b) Overlay of *galacto*‐ configured aziridine **4** (teal) and *gluco*‐configured azide‐tagged epoxide **1** (grey) demonstrating rotation of the O6 hydroxyl of **4**.

To further understand the binding of *galacto‐*configured cyclophellitol aziridines with rhGBA, a co‐crystal structure with *galacto*‐configured **4** was obtained at 1.80 Å. The resulting complex revealed that **4** covalently modifies the catalytic nucleophile of GBA in an almost identical fashion to *gluco*‐configured reagents **1** and **2**. Specifically, **4** reacts with Glu340 via trans‐diaxial ring opening of the aziridine trap to form a covalent complex in the ^4^C_1_ conformation with a bond length of 1.45 Å (Figure 4a). The key difference between this *galacto‐*configured complex and the *gluco*‐configured analogues is the axial conformation of the O4 hydroxyl, which is still able to form a hydrogen bond with Asp127. Indeed, the hydrogen bonding network of **4** is very similar to that of *gluco*‐configured **1** and **2**, with both O3 and O4 able to form hydrogen bonds to Asp127 regardless of whether the sugar is *gluco‐* or *galacto‐*configured. The only notable difference is the absence of a hydrogen bond between the O4 hydroxyl and Trp381, presumably owing to its axial conformation.

This led us to ponder why *galacto*‐aziridines appear to be reasonably potent inhibitors of GBA whereas the equivalent O6‐modified *galacto‐*epoxides are essentially unreactive (as reported by Marques et al., 2017). Overlay of *galacto*‐aziridine **4** and *gluco*‐epoxide **1** provides an immediate clue: the O6 hydroxyl of *galacto*‐aziridine **4** has rotated such that it points ‘downwards’, perhaps to avoid steric clash with the axial *galacto*‐O4, (Figure 4b). Thus, one possible explanation for the cross‐reactivity of *galacto*‐aziridines but not O6‐substituted *galacto*‐epoxides, is the requirement to displace the O6‐hydroxyl for covalent binding which would not be possible were the O6‐substituted. Therefore, it appears that the placement of the reporter group is important in controlling the selectivity and cross‐reactivity of cyclophellitol‐based ABPs. In combination with the ABP labelling assays of rhGBA, this co‐crystal structure serves as a caution when assuming similar configurational specificity of cyclophellitol epoxides and aziridines, which evidently does not always translate to similar glycosidase specificity.

### BODIPY‐tagged cyclophellitol epoxide (5) in complex with GBA inactivated with *N*‐acyl aziridine (3)

**Figure 5:**
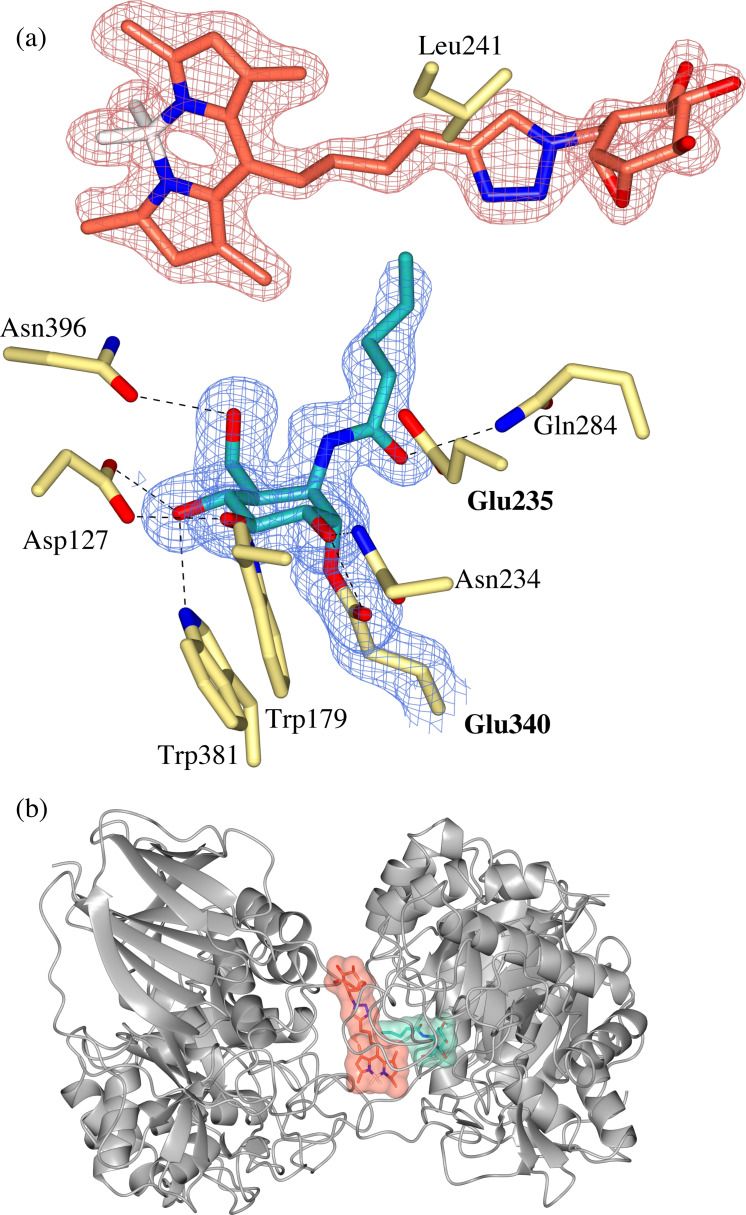
(a) Observed electron density for binding of *N*‐acyl aziridine **3** in the active site of rhGBA and intact BODIPY‐tagged ABP **5** at dimer interface. Maximum‐likelihood/σA weighted (2F_o_–F_c_) electron density map contoured to 1.0 σ (a=1.29 e^−^/Å^3^) (b) Ribbon diagram of rhGBA dimer shows binding of ABP **5** in a hydrophobic cavity at the dimer interface (red surface), with *N*‐acyl **3** bound in the active site (blue surface).

In addition to a previously reported structure of rhGBA in complex with the O6−Cy5 tagged cyclophellitol epoxide ABP **9**,^[27]^ a serendipitous co‐complex of O6−BODIPY tagged ABP **5** and *N*‐acyl aziridine **3** was obtained in this study by accidental soaking of rhGBA crystals in complex *N*‐acyl aziridine **3** with additional ABP **5**. In the resulting co‐complex, clear electron density was observed for **3** bound covalently to Glu340, with its ring‐opened *N*‐acyl chain bound in the narrow active site cavity flanked by Gln283 and Tyr313 (Figure 5a). Additionally, unambiguous electron density for an intact molecule of ABP **5** was observed, allowing the full ABP to be modelled at the surface of molecule A of the crystallographic dimer, (Figure 5b). Specifically, the BODIPY tag binds at the dimer interface in a hydrophobic cavity formed by Leu241, Tyr244, Pro245, Phe246 and Tyr313, which we reported previously to accommodate the Cy5 tag of ABP **9**.^[27]^ The unreacted cyclophellitol of **5** sits exposed at the surface of the protein, making no hydrogen bonding interactions with the enzyme; however, the hydrophobic face of the cyclophellitol unit lies above the exposed side chain of Leu241 (Figure 5a). Whilst the binding of ABP **5** could be considered artefactual, it is notable that very simple torsional rotation of the linker allows the linker and cyclophellitol unit to be placed in the active site, putting the epoxide warhead in perfect super‐position with the trapped aziridine without any movement of the BODIPY group itself. In combination with the previously reported structure of rhGBA in complex with a Cy5 tagged ABP **9**,^[27]^ this ABP **5** co‐complex provides further evidence for a unique binding mode of this hydrophobic cavity, which has recently been exploited for the binding of a novel class of pyrrozopyrazine activators with chaperoning potential.^[38]^

GBA is notable for its tolerance, indeed preference, for O6‐substituted reagents which exhibit increased specificity for GBA over other β‐glucosidases, including non‐lysosomal GBA (GBA2) and generic GH1 β‐glucosidases.^[27]^ GBA also favours imino‐sugar inhibitors and cyclophellitol aziridines extended at the aziridine nitrogen position. Considering these preferences and the structural information provided by the co‐complex of **3** and **5**, we suggested that binding of the O6‐substituent and aziridine *N*‐functionalization are structurally exclusive and may reflect the enzymes specificity for a lipid substrate with two acyl tails. This led us to ponder whether a new generation of bi‐functional aziridine ABPs, which are functionalised at the O6‐position and aziridine nitrogen, may exhibit further improvements in potency and selectivity for GBA. Consequently, the O6−Cy5 tagged *N*‐octyl bifunctional aziridine ABP **6** was synthesised and its GBA activity and selectivity were compared to the parent Cy5 tagged cyclophellitol aziridine ABP **8** and cyclophellitol epoxide ABP **9**.

### Cy5 Tagged Bi‐Functional Cyclophellitol Aziridine (6)

#### Synthesis of Cy5 and *N*‐octyl bifunctional cyclophellitol aziridine (6)

**Scheme 2:**
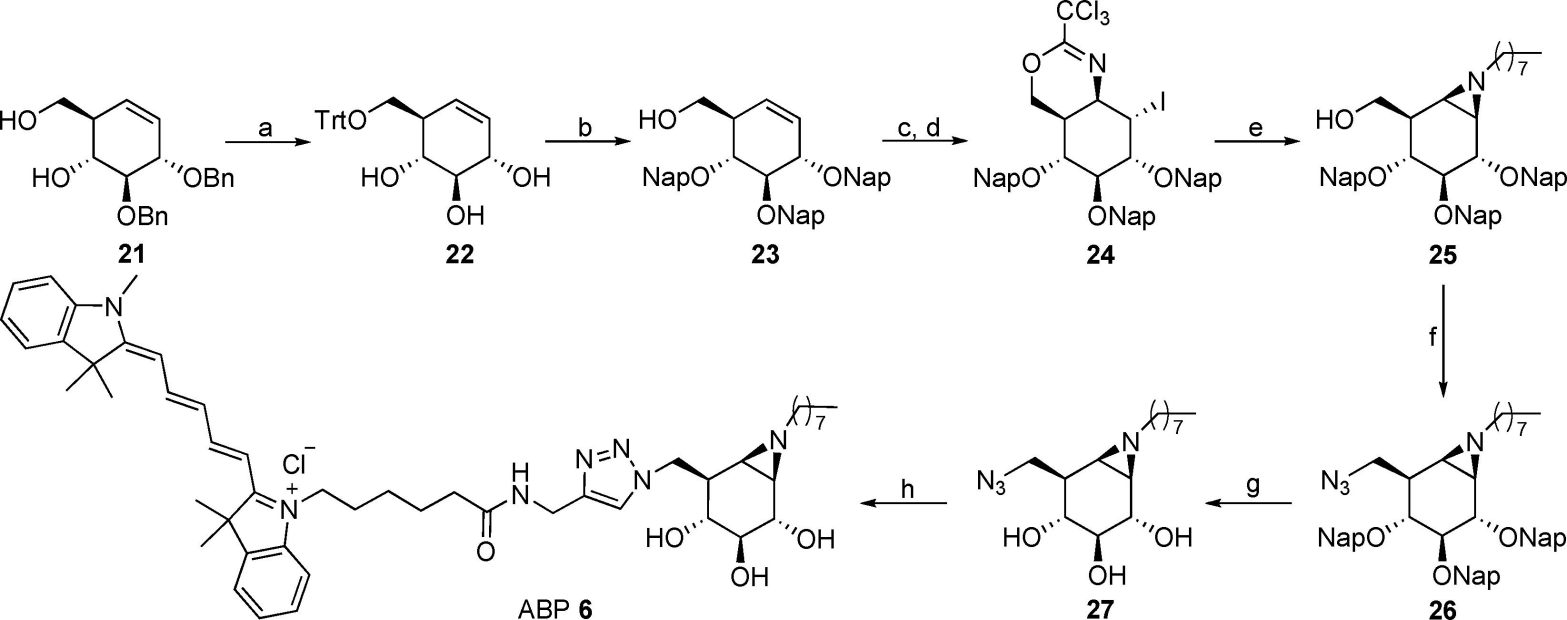
Synthesis of ABP **6**. Reagents and conditions: a) *i*) BCl_3_, DCM, −78 °C, 2 h; *ii*) TrCl, Et_3_N, DMAP, DMF, rt, 19 h, 30 % over two steps; b) *i*) NapBr, NaH, TBAI, DMF, 0 °C‐rt, 5 h; *ii*) TsOH, DCM/MeOH (1/1), rt, overnight, 73 % over two steps; c) Cl_3_CCN, DBU, DCM, rt, overnight; d) NIS, CHCl_3_, rt, 17 h, 40 % over two steps; e) *i*) HCl, DCM/MeOH (1/1), rt, overnight, then Amberlite IRA‐67, 20 h; *ii*) 1‐iodooctane, K_2_CO_3_, DMF, 80 °C, 16 h, 35 % over two steps; f) *i*) TsCl, Et_3_N, 1‐methyl imidazole, DCM, rt, 28 h; *ii*) NaN_3_, DMF, 50 °C, 40 h, 68 % over two steps; g) DDQ, DCM/H_2_O (10/1), rt, 24 h, 66 %; h) Cy5‐alkyne, CuSO_4_, NaAsc, DMF, rt, overnight, 30 %.

In this work, we developed a new synthetic route towards 6‐azido octyl aziridine **27** following a key 2‐napthylmethyl ether (Nap) protecting group strategy (Scheme 2). Briefly, starting intermediate **21** was synthesized in nine steps from d‐xylose based on chemistry developed by Madsen and co‐workers.[^67^, ^68^] Benzyl deprotection was not compatible with the required azide functionality and electrophilic aziridine, therefore, debenzylation of **21** with boron trichloride (BCl_3_) and selective tritylation of the primary alcohol afforded intermediate **22**. The secondary alcohols were protected as Nap ethers followed by detritylation of the primary alcohol to afford **23**. Treatment of **23** with trichloroacetonitrile yielded a primary imidate intermediate, which was treated with *N*‐iodosuccinimide (NIS) to stereospecifically afford the cyclic imidate **24**. Acidolysis and base treatment of **24** resulted in the formation of a free aziridine, which was then alkylated with an octyl linker to form compound **25**. Tosylation of the primary alcohol of **25** followed by azide substitution resulted in **26**. Napthylmethyl ethers were then removed by DDQ to afford compound **27**, which was finally coupled with Cy5 using click chemistry to yield ABP **6** (Scheme 2).

#### Cy5‐tagged bifunctional cyclophellitol aziridine (6)

**Figure 6:**
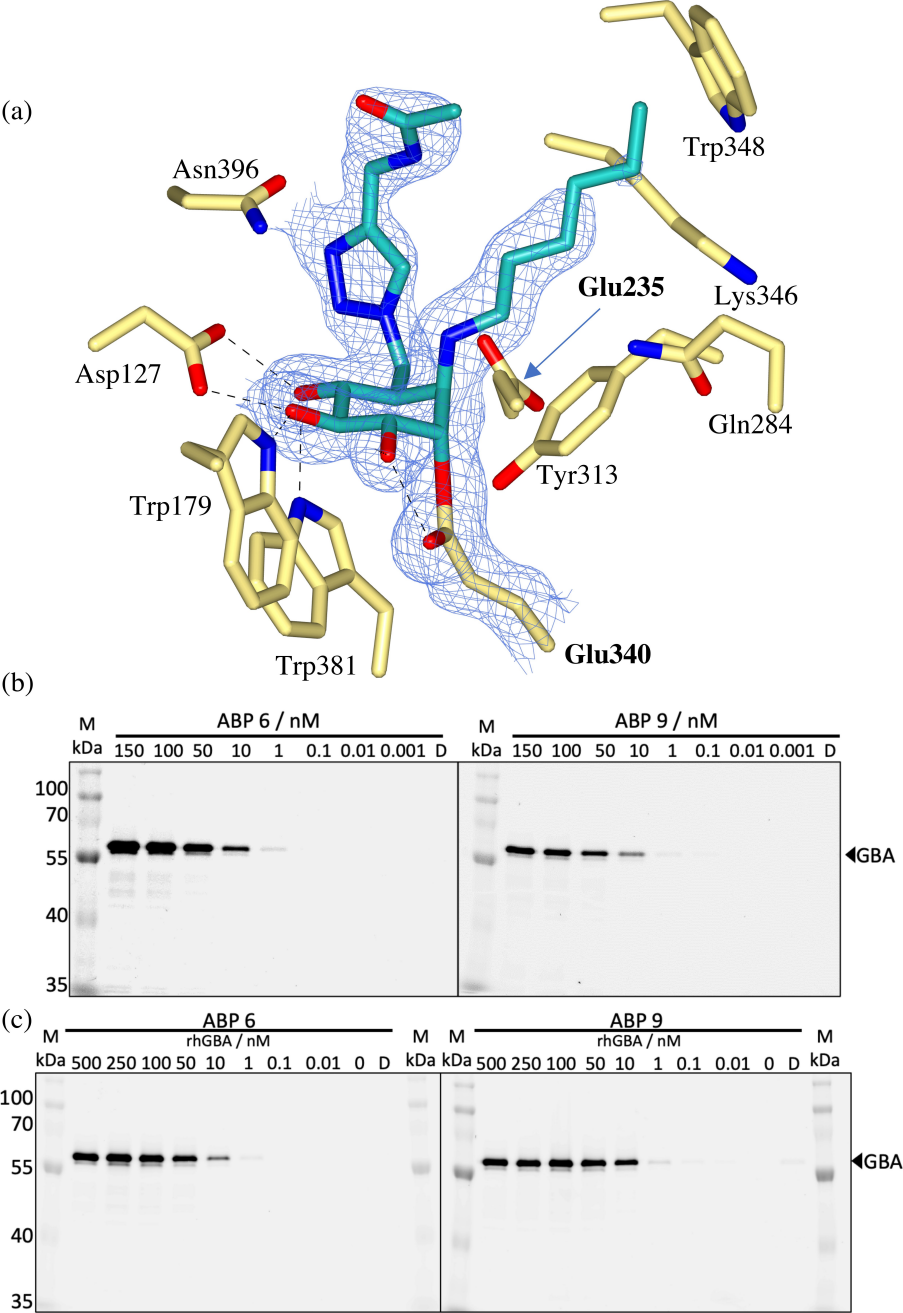
(a) Observed electron density for ABP **6** bound covalently to the catalytic nucleophile (Glu340) of rhGBA by trans‐diaxial ring opening of the *N*‐alkyl aziridine warhead. Maximum‐likelihood/σA weighted (2F_o_–F_c_) electron density map contoured to 1.0 σ (a=1.31 e^−^/Å^3^). (b) Labeling of rhGBA (200 nM) with decreasing concentrations of ABP **6** or ABP **9** (150–0.001 nM) at 37 °C for 30 mins followed SDS‐PAGE separation. (c) Incubation of ABP **6** or ABP **9** (150 nM) with decreasing concentrations of rhGBA (500–0.01 nM) followed by SDS‐PAGE analysis. Fluorescently labelled rhGBA visualized by Cy5 fluorescent readout. D=denatured protein sample.

To investigate the accommodation of the two functionalities of ABP **6** by GBA, a co‐crystal structure in complex with bi‐functional ABP **6** was obtained at 1.80 Å resolution, demonstrating covalent binding of the cyclophellitol aziridine to the catalytic nucleophile of GBA (Figure 6a). Specifically, the reacted cyclophellitol adopts the expected ^4^C_1_ chair conformation, with a covalent bond length of 1.47 Å to Glu340. Furthermore, unambiguous electron density for the ring opened *N‐*alkyl aziridine warhead was observed, allowing the first 5 carbons of the *N‐*octyl chain to be modelled. This was sufficient to establish binding of the *N*‐alkyl chain to the narrow active site channel formed by Gln284, Tyr313, Lys346 and Trp348, which is consistent with the complex of *N*‐acyl aziridine **3**. In fact, the *N*‐alkyl chain of ABP **6** extends through this pocket towards the surface of the protein, which may provide some indication into the binding of the fatty acid portion of the natural GlcCer substrate which is thought to project out from the protein and interact with the lipid bilayer.^[30]^ Unfortunately, whilst sufficient electron density for the O6‐triazole linker and subsequent amide group was observed, the Cy5 tag could not be modelled. Nevertheless, the O6‐triazole linker was found to bind in the hydrophobic cavity formed by Trp348, Phe246 and Tyr313, which we reported previously to accommodate the triazole linker of ABP **9**.^[27]^ Additionally, this binding cavity extends towards the broader hydrophobic allosteric site at the dimer interface where we reported the BODIPY tag of ABP **5** to bind.

#### Activity‐based labelling of rhGBA with ABP 6 and ABP 9

Activity‐based labelling of rhGBA (produced in an insect‐baculovirus expression vector system^[58]^) with bi‐functional ABP **6** was performed and compared to labelling by its mono‐functionalized epoxide derivative ABP **9**. Firstly, in solution labelling of excess but constant rhGBA (200 nM) was performed in the presence of decreasing ABP concentration (150–0.001 nM), demonstrating clear concentration dependent labelling with a gel‐detection limit of 1 nM for ABP **6** and 0.1 nM for ABP **9** (Figure 6b). Secondly, labelling assays in which ABP **6** and ABP **9** (150 nM) were incubated with decreasing rhGBA concentrations (500–0.01 nM) were performed to further demonstrate the concentration dependent labelling down to 1 nM rhGBA with ABP **6** and 0.1 nM rhGBA with ABP **9**, (Figure 6c). These in gel detection limits are concordant across both assays and demonstrate that ABP **9** exhibits ca. 10‐fold increase in potency.

#### *In vitro* activity and selectivity of Cy5 biofunctionalized cyclophellitol aziridine ABP 6

To further investigate the potency and selectivity of ABP **6**, *in vitro* activity assays against GBA and two other related β‐ and α‐glucosidases (GBA2 and GAA) were performed and compared to ABP **9**.

ABP **6** and ABP **9** were pre‐incubated with recombinant human GBA (rhGBA, Imiglucerase), human GBA2 (from lysates of GBA2 overexpressed cells) and recombinant human GAA (rhGAA, Myozyme) for 3 hr followed by enzymatic activity measurement using 4‐methylumbelliferyl‐β‐ and α‐glucosides as fluorogenic substrates. As shown in Table 1, ABP **6** proved to be a nanomolar inhibitor of GBA (with an apparent IC_50_ value of 53 nM) and is inactive toward GBA2 and GAA (apparent IC_50_ values >100 μM), thus exhibiting comparable selective inhibition of GBA (IC_50_ ratio >10^3^ for both GBA2/GBA and GAA/GBA) as we reported previously for ABP **9**.^[27]^

**Table 1:** Apparent IC_50_ values for *in vitro* inhibition of rhGBA, rhGAA and GBA2 from overexpressed cell lysates by ABP **6** and **9**. Error ranges depict standard deviations from technical duplicates.

Compound	*In vitro* rhGBA IC_50_ (nM)	*In vitro* GBA2 (HEK293T lysate) IC_50_ (nM)	*In vitro* rhGAA IC_50_ (nM)
**ABP 6**	53.1±2.65	> 10^5^	> 10^5^
**ABP 9** ^[27]^	3.20±0.17	412×10^3^± 10.1×10^3^	> 10^5^

**Figure 7:**
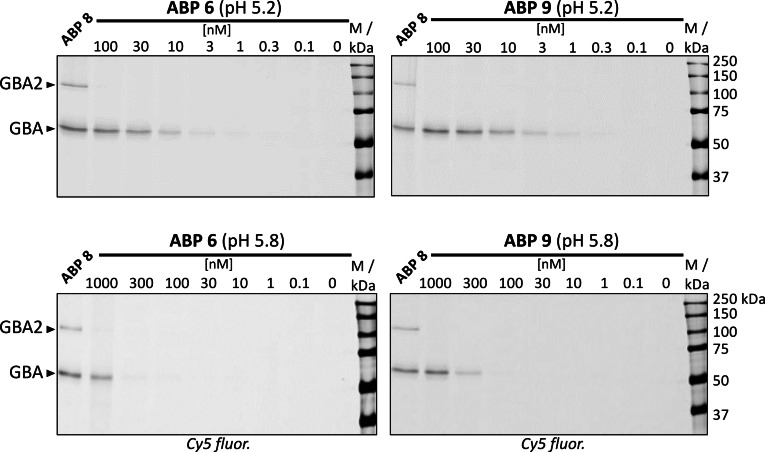
Fluorescent labeling of mouse brain lysate (25 μg total protein) with different concentrations of bi‐functional ABP **6** and mono‐functional ABP **9** at pH 5.2 (upper panels) or pH 5.8 (lower panels) after incubation for 30 min at 37 °C. Labeling by broad‐spectrum β‐glucosidase ABP **8** is shown for comparison (100 nM, pH 5.2 or pH 5.8, 30 min, 37 °C).

We next investigated their labeling efficiency and selectivity toward GBA in mouse brain lysate at pH 5.2 (containing 0.2 % taurocholate and 0.1 % Triton‐100) and pH 5.8 as respective optimal conditions for GBA and GBA2 activities (Figure 7). As expected, ABP **6** and **9** selectively labeled GBA in a concentration‐dependent manner under pH 5.2 (upper panels), with significant labeling observed at 10 nM ABP. Under pH 5.8 (lower panels), the labeling efficiency of both ABPs towards GBA decreased and significant labeling can only be observed at 1000 nM (ABP **6**) or 300 nM (ABP **9**). Importantly, no labelling of GBA2 was observed up to 1000 nM, showing good GBA selectivity. For comparison, broad‐spectrum β‐glucosidase ABP **8** efficiently labelled both GBA and GBA2 at 100 nM under both pHs.

#### *In situ* labeling of GBA and GBA2 in living cells by ABP 6, 8 and 9

**Figure 8:**
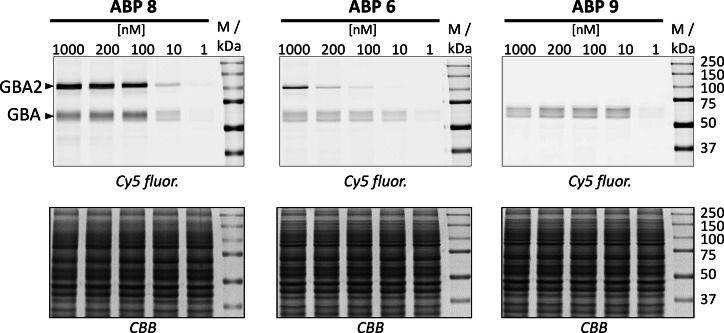
*In situ* labeling of GBA and GBA2 in HEK293T cells with ABP **6**, ABP **8** and ABP **9** at varying concentrations at 37 °C for 24 h, followed by SDS‐PAGE separation and fluorescent readout (top panel). *CBB*, Coomassie Brilliant Blue staining (bottom panel).

To further evaluate the selectivity of ABP **6**, **8** and **9**, *in situ* labeling of GBA and GBA2 in living cells were investigated. HEK293T cells containing endogenous GBA and overexpressed GBA2 were treated with the ABPs at different concentrations (1–1000 nM) for 24 hr. Cells were then washed, lysed and the fluorescence was visualized by gel‐based ABPP (Figure 8). Treatment with broad spectrum ABP **8** resulted in unbiased labeling of GBA and GBA2 at 10 nM, with labeling of both enzymes reaching saturation at 100 nM after 24 h incubation. In comparison, selective labeling of GBA in ABP **6** treated cells was observed at 10 nM, with some GBA2 labelling observed at higher probe concentrations (100 nM). More selective labeling of GBA was achieved with ABP **9**, which did not label GBA2 even at the highest concentration of probe applied (1000 nM).

Therefore, despite demonstrating that the O6‐ and aziridine nitrogen substituents of ABP **6** are structurally exclusive and are accommodated by GBA in two unique active site clefts, ABP **6** exhibits no further improvements in potency or selectivity for GBA over the O6 mono‐functionalized ABPs. Nevertheless, this bi‐functional ABP remains a nanomolar inhibitor of GBA which provides future opportunities for ABP development through modification of both the O6‐ and aziridine nitrogen substituents. This work also exemplifies the complexity of ABP development.

## Conclusion

Tagged cyclophellitols offer a powerful activity‐based protein profiling approach for the visualization and quantification of specific enzymatic activities. Here, we report the design, synthesis, and structural analysis of a range of cyclophellitol epoxide and aziridine inactivators and activity‐based probes (ABPs) for human β‐glucocerebrosidase (GBA). These studies not only demonstrate the mechanism‐based mode of action of these compounds as covalent inactivators, but also highlight binding of *N*‐functionalized aziridines to the active site cleft. The cyclophellitol‐based inhibitors subsequently served as scaffolds for the development of ABPs; the O6‐fluorescent tags of which bind to an allosteric site at the dimer interface. In light of the accommodation of *N‐*functionalized aziridines and O6‐substituents by GBA, we synthesized a bi‐functional O6−Cy5 *N*‐octyl aziridine ABP which we hoped would offer a more powerful imaging agent. Whilst we structurally validated that the O6‐ and aziridine functionalities are structurally exclusive and bind in two distinct active site clefts, this bi‐functional ABP showed no benefit in potency or selectivity over the mono‐functionalized ABPs. Nevertheless, this study provides fundamental insight into ABP reactivity, specificity, and conformation with a tale of caution on ABP cross‐reactivity when assuming similar glycosidase specificity of configurationally isomeric cyclophellitol epoxides and aziridines. We envisage these inhibitors and ABPs will serve useful in the study of GBA in relation to Gaucher Disease and inform the design of next‐generation inhibitors and probes.

## Experimental Section

### Synthesis: General experimental details

General synthetic details can be found in the Supporting Information (page 3).

### Experimental procedures and characterization data of products

Inhibitors **1**,^[28]^ **2**,^[68]^ **3**,^[57]^ **4**,^[63]^ and ABPs **5** ^[28]^ and **8** ^[66]^ were synthesized according to previously published procedures and their spectroscopic data are in agreement with those reported previously. ABPs **6** and **7** were synthesised and characterised according to the procedures outlined in the Supporting Information (pages 3–7).

### Biochemical methods: General experimental details

Recombinant human GBA (rhGBA, imiglucerase, Cerezyme®) and GAA (rhGAA, alglucosidase alfa, Myozyme) were obtained from Sanofi Genzyme (Cambridge, MA, USA). rhGBA was also produced in an insect‐baculovirus expression vector system (BEVS) and purified according to previously published procedures.^[58]^ Other general biochemical details can be found in the Supporting Information (page 7).

### *In vitro* activity of ABP 6 on rhGBA, GBA2 and rhGAA

*In vitro* apparent IC_50_ measurements of ABP **6** with rhGBA and rhGAA were determined using the fluorogenic substrate methods described previously.^[27]^ For *in vitro* apparent IC_50_ measurements of GBA2, 8 volumes of cell lysates (4 μg total protein/μL) containing overexpressed human GBA2 were firstly pre‐incubated with 1 volume of MDW941 (100 nM final concentration, 0.5 % (v/v DMSO)) for 30 min at 37 °C to selectively inhibit GBA activity. Lysates were then incubated with 1 volume of ABP **6** at various concentrations for 3 h at 37 °C, before subsequent enzymatic assay for GBA2 activity as described earlier.[^27^, ^69^] All assays were performed in duplicate sets, each with 3 technical replicates at each inhibitor concentration. DMSO concentration was kept at 0.5 %–1 % (v/v) in all assays during incubation with compounds. *In vitro* apparent IC_50_ values were calculated by fitting data with [inhibitor] vs. response‐various slope (four parameters) function using Graphpad Prism 7.0 software. Average values and standard deviations were calculated from the two sets.

### Time‐dependent labelling assays of rhGBA with *galacto*‐ABP 7 and *gluco*‐ABP 8

rhGBA produced in BEVS^[58]^ was prepared at 700 nM in 150 mM McIlvaine buffer pH 5.2 (containing 0.1 % (v/v) Triton X‐100 and 0.2 % (w/v) sodium taurocholate) and ABP **7** or ABP **8** were added to 150 nM. The reactions were incubated at 37 °C and aliquots were taken at 2, 5, 10, 30 and 60 mins. The aliquots were immediately denatured with Laemmli (x3) sample buffer by heating at 95 °C for 5 minutes. The samples were resolved by electrophoresis in 10 % SDS‐PAGE gels, running at 200 V for ∼50 minutes. Wet slab gels were scanned on fluorescence using an AmershamTyphoon 5 Imager (GE Healthcare) with λ_EX_ 635 nm; λ_EM_ >665 nm.

### Titration of ABP 6 and ABP 9 with rhGBA

rhGBA produced in BEVS^[58]^ was diluted to 200 nM in 150 mM McIlvaine buffer pH 5.2 (containing 0.1 % (v/v) Triton X‐100 and 0.2 % (w/v) sodium taurocholate) and ABP **6** or ABP **9** were added to 150, 100, 50, 10, 1, 0.1, 0.01 or 0.001 nM final concentration. The reactions were incubated at 37 °C for 30 mins and denatured with Laemmli (x3) sample buffer at 95 °C for 5 minutes. The samples were resolved by electrophoresis in 10 % SDS‐PAGE gels, running at 200 V for ∼50 minutes. Wet slab gels were scanned on fluorescence using an Amersham Typhoon 5 Imager (GE Healthcare) with λ_EX_ 635 nm; λ_EM_ >665 nm.

### rhGBA Titration with ABP 6 and ABP 9

rhGBA produced in BEVS^[58]^ was prepared at 500, 250, 100, 50, 10, 1, 0.1 and 0.01 nM in 150 mM McIlvaine buffer pH 5.2 (supplemented with 0.1 % (v/v) Triton X‐100 and 0.2 % (w/v) sodium taurocholate). ABP **6** or ABP **9** were added to 150 nM final concentration and the reactions were incubated at 37 °C for 30 mins. The samples were denatured with Laemmli (x3) sample buffer by heating at 95 °C for 5 minutes and resolved by electrophoresis in 10 % SDS‐PAGE gels, running at 200 V for ∼50 minutes. Wet slab gels were scanned on fluorescence using an Amersham Typhoon 5 Imager (GE Healthcare) with λ_EX_ 635 nm; λ_EM_ >665 nm.

### Fluorescent labeling of lysates and SDS‐PAGE analysis

Mouse brain lysate (25 μg total protein per sample) was diluted with 150 mM McIlvaine buffer pH 5.2 (with 0.1 % (v/v) Triton X‐100 and 0.2 % (w/v) sodium taurocholate) or pH 5.8 to a final 10 μL volume and labeled with different concentrations of ABP **6** or **9** (diluted with McIlvaine buffer at matching pH to a final 5 μL volume) at 37 °C for 30 min. Fluorescent labeling with broad‐spectrum ABP **8** was performed at 100 nM ABP concentration at 37 °C for 30 min at pH 5.2 (with 0.1 % (v/v) Triton X‐100 and 0.2 % (w/v) sodium taurocholate) or pH 5.8 respectively. Samples were then denatured with 4 μL Laemmli (5x) sample buffer and heated at 98 °C for 5 minutes. Proteins were resolved by electrophoresis in 10 % SDS‐PAGE gels, running at a constant of 90 V for 30 minutes followed by 120 V for approximately 60 minutes. Wet slab gels were scanned on fluorescence using a Typhoon FLA9500 Imager (GE Healthcare) using λ_EX_ 635 nm; λ_EM_ >665 nm and images were processed using ImageLab 5.2.1 (BioRad).

### *In situ* labeling of HEK 293T cells and SDS‐PAGE analysis

Labelling of HEK293T cells, containing endogenous GBA and overexpressed GBA2, with ABPs **6, 8** and **9** was conducted based on previously described methods^[69]^ as outlined in the Supporting Information (page 7).

### Structural biology

#### Crystallisation of recombinant GBA (Imiglucerase)

Recombinant human GBA (rhGBA, Imiglucerase, Cerezyme®) supplied by Sanofi‐Genzyme (Cambridge, MA, USA) was dialyzed overnight against phosphate buffered saline (PBS) (pH 7.0) and buffer exchanged into 50 mM 2‐(N‐morpholino)ethanesulfonic acid (MES), 100 mM NaCl pH 6.6 using an S75 16/600 column before partially de‐glycosylating with *N‐*glycosidase F according to previous procedures.^[30]^ rhGBA was concentrated to 10 mg/mL and crystallised by hanging drop vapor diffusion in 24‐well plates using previously reported conditions.^[30]^

#### Co‐crystal complexes with imiglucerase (1, 3, 4 and 5)

Co‐crystal structures of Imiglucerase in complex with **1, 3, 4** and **5** were obtained either by adding a small amount of solid compound directly to a drop containing rhGBA crystals or by adding 1 μl of the compound dissolved in mother liquor [1.0 M (NH_3_)_2_SO_4_, 0.17 M guanidine HCl, 0.02 M KCl, 0.1 M sodium acetate pH 4.6] to the crystallisation drop and soaking for 30 minutes.

#### Production and crystallisation of recombinant GBA from BEVS

Recombinant human GBA (rhGBA) was produced in BEVS and purified according to previously published procedures.^[58]^ rhGBA was subsequently crystallised in a 48‐well MRC sitting‐drop vapour‐diffusion format using previously reported 4‐(2‐hydroxyethyl)‐1‐piperazineethanesulfonic acid (HEPES) containing conditions.^[58]^

#### Co‐crystal complexes with rhGBA from BEVS (2 and 6)

Co‐crystal complexes of **2** and **6** were obtained by soaking unliganded rhGBA crystals (produced in BEVS) overnight in mother liquor [0.2 M sodium sulfate, 0.25 M HEPES pH 7.0, 14 % (v/v) PEG 3350] spiked with 0.5 mM **2** or 2 mM ABP **6** and 10 % DMSO.

#### Data collection, structure solution and refinement

All crystals were transferred to a cryoprotectant solution containing 15 % ethylene glycol or 20–25 % glycerol before flash freezing in liquid nitrogen for data collection. Data for all co‐crystal complexes were collected at the i02, i03 and i04 beamlines of the Diamond Light Source (DLS) UK, and processed using XIA2^[70]^ and AIMLESS[^71^, ^72^] data reduction pipeline in the CCP4i2 suite.^[73]^ The previous GBA PDB 2NT0^[74]^ was used to solve the structure of unliganded Imiglucerase by molecular replacement using MOLREP.^[75]^ Coordinates of unliganded Imiglucerase were used for direct determination of the ligand complexes of **1, 3, 4** and **5** using REFMAC.^[76]^ Ligand complexes of **2** and **6** with rGBA (produced in BEVS) were solved by molecular replacement using MOLREP with the previous unliganded structure PDB 6TJK^[58]^ as the homologous search model.

Refinement of all structures was performed using REFMAC^[76]^ followed by several rounds of manual model building with COOT.[^77^, ^78^] Idealized coordinate sets and refinement dictionaries for each ligand were generated using JLIGAND^[79]^ or ACEDRG.[^80^, ^81^] Conformation of all sugars were validated using Privateer^[82]^ and all structures were validated using MolProbity^[83]^ and the wwPDB Validation service (validate‐rcsb‐1.wwpdb.org/) prior to deposition. Data collection and refinement statistics are summarised in Table S1 and Table S2 (Supporting Information). Crystal structure figures were generated in CCP4 mg.^[84]^

## Conflict of interest

The authors declare no conflict of interest.

## Supporting information

As a service to our authors and readers, this journal provides supporting information supplied by the authors. Such materials are peer reviewed and may be re‐organized for online delivery, but are not copy‐edited or typeset. Technical support issues arising from supporting information (other than missing files) should be addressed to the authors.

Supporting Information
