# Supplementary material for: Design, Synthesis and Structural Analysis of Glucocerebrosidase Imaging Agents
Source: Chemistry. 2021 Oct 29;27(66):16377–88. doi: 10.1002/chem.202102359 (PMC9298352; doi:10.1002/chem.202102359)
Supplement: Supplementary file 1 — Supporting Information [file CHEM-27-16377-s001.pdf]

Chemistry–A European Journal

Supporting Information

Design, Synthesis and Structural Analysis of Glucocerebrosidase Imaging Agents

Rhianna J. Rowland, Yurong Chen, Imogen Breen, Liang Wu, Wendy A. Offen, Thomas J. Beenakker, Qin Su, Adrianus M. C. H. van den Nieuwendijk, Johannes M. F. G. Aerts, Marta Artola, Herman S. Overkleeft,* and Gideon J. Davies*

Supporting information

Contents

Experimental

Synthesis: General Experimental Details……………………………………………..…………3

Synthesis: Experimental Procedures and Characterisation of Products………………………..…3

Synthesis of ABP 6……………………………………………………………………..3

Synthesis of ABP 7……………………………………………………………………..5

Biochemical Methods: General Experimental Details…………………….………………………7

Biochemical Methods: Experimental Procedures…………………………………………………7

In situ Labeling of HEK 293T cells……………………………………………………....7

Biochemical Methods: pH Dependent activity-based labelling of rhGBA………………...7

Crystallography

Table S1: Data Collection and Processing...8

Table S2: Structure Solution and Refinement..9

NMR Spectra

Figure S2: ^{1}H-NMR and ^{13}C-NMR spectra of **22** in $CDCl_3$..10

Figure S3: ^{1}H-NMR and ^{13}C-NMR spectra of **23** in $CDCl_3$..11

Figure S4: ^{1}H-NMR and ^{13}C-NMR spectra of **24** in $CDCl_3$..12

Figure S5: ^{1}H-NMR and ^{13}C-NMR spectra of **25** in $CDCl_3$..13

Figure S6: ^{1}H-NMR and ^{13}C-NMR spectra of **26** in $CDCl_3$..14

Figure S7: ^{1}H-NMR and ^{13}C-NMR spectra of **27** in MeOD..15

Figure S8: ^{1}H-NMR and ^{13}C-NMR spectra of **ABP 6** in MeOD..16

Figure S9: ^{1}H-NMR and ^{13}C-NMR spectra of **12** in $CDCl_3$..17

Figure S10: ^{1}H-NMR and ^{13}C-NMR spectra of **13** in $CDCl_3$..18

Figure S11: ^{1}H-NMR and ^{13}C-NMR spectra of **15** in $CDCl_3$..19

Figure S12: ^{1}H-NMR and ^{13}C-NMR spectra of **16** in $CDCl_3$..20

Figure S13: ^{1}H-NMR and ^{13}C-NMR spectra of **17** in $CDCl_3$..21

Figure S14: ^{1}H-NMR and ^{13}C-NMR spectra of **18** in $CDCl_3$..22

Figure S15: ^{1}H-NMR and ^{13}C-NMR spectra of **19** in D_2O..23

References……………………………………………………………………..24

Experimental Section

Synthesis: General Experimental Details

All reagents were of a commercial grade and were used as received unless stated otherwise. Polymer-bound PPh_3 (100-200 mesh, 3.0 mmol/g loading) was purchased from Sigma-Aldrich. Dichloromethane (DCM), chloroform ($CHCl_3$), toluene, tetrahydrofuran (THF) and *N*,*N*-dimethylformamide (DMF) were stored over 4 Å molecular sieves, which were dried *in vacuo* before use. Triethylamine was dried over KOH and distilled before using. All reactions were performed under an argon atmosphere unless stated otherwise. Solvents used for flash column chromatography were of pro analysis quality. Reactions were monitored by analytical thin-layer chromatography (TLC) using Merck aluminum sheets pre-coated with silica gel 60 with detection by UV absorption (254 nm) and by spraying with a solution of $(NH_4)_6Mo_7O_{24}\cdot H_2O$ (25 g/L) and $(NH_4)_4Ce(SO_4)_4\cdot H_2O$ (10 g/L) in 10% sulfuric acid followed by charring at ~150 ˚C or by spraying with an aqueous solution of $KMnO_4$ (7%) and K_2CO_3 (2%) followed by charring at ~150 °C. Column chromatography was performed manually using either Baker or Screening Device silica gel 60 (0.04 - 0.063 mm) or a Biotage Isolera™ flash purification system using silica gel cartridges (Screening devices SiliaSep HP, particle size 15-40 μm, 60A) in the indicated solvents. ^1H NMR and ^{13}C NMR spectra were recorded on Bruker DMX-600 (600/150 MHz), Bruker AV-400 (400/101 MHz), Bruker AV-500 (500/126 MHz) and Bruker AV-850 (850/214 MHz) spectrometers in the given solvent. Chemical shifts are given in ppm relative to the chloroform residual solvent peak or tetramethylsilane (TMS) as internal standard. Coupling constants are given in Hz. All given ^{13}C spectra are proton decoupled. The following abbreviations are used to describe peak patterns when appropriate: s (singlet), d (doublet), t (triplet), qt (quintet), m (multiplet), br (broad), ar (aromatic), app (apparent). 2D NMR experiments (HSQC, COSY and NOESY) were carried out to assign protons and carbons of the new structures and assignation follows the general numbering shown in cyclohexene 10. High-resolution mass spectra (HRMS) of intermediates were recorded with a LTQ Orbitrap (Thermo Finnigan) and final compounds were recorded with an apex-QE instrument (Bruker). LC/MS analysis was performed on an LCQ Advantage Max (Thermo Finnigan) ion-trap spectrometer (ESI+) coupled to a Surveyor HPLC system (Thermo Finnigan) equipped with a C18 column (Gemini, 4.6 mm x 50 mm, 3 μm particle size, Phenomenex) equipped with buffers A: H_2O, B: acetonitrile (MeCN) and C: 1% aqueous TFA, or an Agilent Technologies 1260 Infinity LCMS with a 6120 Quadrupole MS system equipped with buffers A: H_2O, B: acetonitrile (MeCN) and C: 100 mM NH_4OAc. For reversed-phase HPLC-MS purifications an Agilent Technologies 1200 series prepLCMS with a 6130 Qudropole MS system was used equipped with buffers A: 50 mM NH_4HCO_3 in H_2O and B: MeCN.

Synthesis: Experimental Procedures and Characterization of Products

Synthesis of ABP 6

(1*R*,2*R*,3*S*,6*R*)-6-((Trityloxy)methyl)cyclohex-4-ene-1,2,3-triol (**22**)

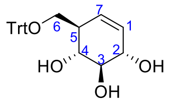

Compound **21** (1.0 g, 2.9 mmol) was dissolved in dry DCM (20 mL) and cooled to -78 °C. BCl_3 (1.0 M in DCM, 15 mL, 15.0 mmol) was added slowly and the mixture was stirred at -78 °C for 2 h. After quenching with MeOH, the solvent was evaporated and the residue was co-evaporated with toluene (3 x) and directly dissolved in dry DMF (15 mL). Then Et_3N (1.0 mL, 7.5 mmol), trityl chloride (1.67 g, 6.0 mmol), and DMAP (18.3 mg, 0.15 mmol) were added and the reaction mixture was stirred at rt for 19 h. The mixture was diluted with H_2O, extracted with EtOAc (2 x), the combined organic layers were washed with water (2 x) and brine, dried with anhydrous Na_2SO_4, filtered and concentrated *in vacuo*. The product was purified by silica gel column chromatography (DCM/MeOH, 50:1→9:1) affording compound **10** (361 mg, 30% over two steps) as a pale-yellow oil. ^1H NMR (400 MHz, $CDCl_3$) δ 7.47 – 7.35 (m, 5H, 5CH Ar), 7.31 – 7.14 (m, 10H, 10CH Ar), 5.53 (dt, *J* = 10.1, 2.4 Hz, 1H, H7), 5.40 (dt, *J* = 10.2, 2.1 Hz, 1H, H1), 4.18 – 4.06 (m, 1H, H2), 3.61 - 3.49 (m, 2H, H3 and H4), 3.25 (dd, *J* = 8.8, 5.4 Hz, 1H, H6a), 3.21 – 3.13 (m, 1H, H6b), 2.50 – 2.42 (m, 1H, H5). ^{13}C NMR (101 MHz, $CDCl_3$) δ 143.9 (3C_q Ar), 129.3 (C1), 128.7 (6CH Ar), 128.0 (6CH Ar), 127.4 (C7), 127.2 (3CH Ar), 87.1 (C_q), 77.7 (C3/C4), 72.3 (C2), 72.3 (C3/C4), 65.1 (C6), 44.4 (C5). HRMS (ESI) m/z: $[M+Na]^+$ calc for $C_{26}H_{26}O_4Na$ 425.17233, found 425.17274.

((1*R*,4*S*,5*R*,6*R*)-4,5,6-Tris(naphthalen-2-ylmethoxy)cyclohex-2-en-1-yl)methanol (**23**)

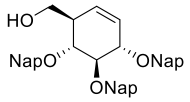

Compound **22** (328 mg, 0.81 mmol) was dissolved in dry DMF (6 mL) and cooled to 0 °C. NaH (60% in mineral, 261 mg, 6.5 mmol) was added to the mixture and stirred at 0 °C for 30 min. Then NapBr (1.08 g, 4.86 mmol) and TBAI (30 mg, 0.08 mmol) were added successively and stirred at 0 °C for 10 min. Then the mixture was warmed to rt and stirred for 5 h. The reaction was quenched with H_2O at 0 °C, diluted with H_2O and extracted with EtOAc (2 x), the combined organic layers were washed with water (2 x) and brine, dried with anhydrous Na_2SO_4, filtered and concentrated *in vacuo*. The crude was then dissolved in a mixture of DCM/MeOH (1/1, 4 mL/4mL). TsOH was added until pH ≈ 2 and the mixture was stirred at rt overnight. Et_3N was added to quench the reaction and the solvent was concentrated *in vacuo*. EtOAc was added and the solution was washed with sat. aq. NH_4Cl, sat. aq. $NaHCO_3$, H_2O and brine, dried over Na_2SO_4, filtered and concentrated *in vacuo*. The product was purified by silica gel column chromatography (pentane/EtOAc, 6:1→3:1) affording compound **23** (342 mg, 73%) as a white solid. ^1H NMR (400 MHz, $CDCl_3$) δ 7.88 – 7.63 (m, 11H, 11CH Ar), 7.53 – 7.34 (m, 10H, 10CH Ar), 5.80 (dt, *J* = 10.1, 2.5 Hz, 1H, H7), 5.57 (dt, *J* = 10.1, 2.0 Hz, 1H, H1), 5.17 (d, *J* = 11.5 Hz, 1H, CH*H* Nap), 5.12 (d, *J* = 4.4 Hz, 2H, CH_2 Nap), 4.87 (s, 2H, CH_2 Nap), 4.83 (d, *J* = 11.3 Hz, 1H, CH*H* Nap), 4.35 (ddd, *J* = 7.8, 3.4, 1.9 Hz, 1H, H2), 3.96 (dd, *J* = 10.1, 7.7 Hz, 1H, H6a), 3.81 – 3.65 (m, 3H, H6b, H3 and H4), 2.59 – 2.53 (m, *J* = 9.4, 3.3 Hz, 1H, H5), 1.78 – 1.49 (br s, 1H, OH). ^{13}C NMR (101 MHz, $CDCl_3$) δ 136.4, 136.0, 135.8, 133.4, 133.4, 133.4, 133.1, 133.1, 133.0 (9C_q Ar), 128.4, 128.4, 128.3, 128.3, 128.2, 128.1, 128.0, 128.0, 127.8, 127.0, 126.9, 126.7, 126.6, 126.3, 126.2, 126.2, 126.1, 126.1, 126.0, 126.0, 125.9 (21CH Ar, C1 and C7), 85.2 (C4), 80.8, 79.0 (C2 and C3), 75.4, 75.3, 72.2 (3CH_2 Nap), 63.4 (C6), 45.9 (C5). HRMS (ESI) m/z: $[M+NH_4]^+$ calc for $C_{40}H_{40}O_4N$ 598.29519, found 598.29479.

((1*R*,4*S*,5*R*,6*R*)-4,5,6-Tris(naphthalen-2-ylmethoxy)cyclohex-2-en-1-yl)methyl-2,2,2-trichloroacetimidate (**S1**)

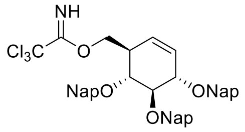

Compound **23** (340 mg, 0.59 mmol) was dissolved in dry DCM (5 mL). Then trichloroacetonitrile (117 µL, 1.17 mmol) and DBU (9 µL, 0.06 mmol) were added and the mixture was stirred at rt overnight. The mixture was concentrated and the product was purified by silica gel column chromatography (pentane/EtOAc, 13:1→7:1) affording intermediate **S1** (302 mg) as a clean oil which was not completely pure and a yield over two steps is provided after the next step. *Note: TLC-analysis showed clean conversion of the reaction, while the product partly decomposed during a purification attempt by column chromatography, forming a less polar impurity which was eluted out together with the product.*

(4a*R*,5*R*,6*S*,7*R*,8*S*,8a*R*)-8-Iodo-5,6,7-tris(naphthalen-2-ylmethoxy)octahydro-2*H*-benzo[d][1,3]oxazin-2-one (**24**)

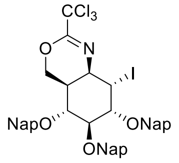

Compound **S1** (300 mg) was co-evaporated with toluene, dissolved in dry $CHCl_3$ (4.5 mL) and cooled down to 0 °C. Then NIS (158 mg, 0.7 mmol) was added and the mixture was stirred at rt for 17 h. The reaction was quenched with sat. aq. $Na_2S_2O_3$ and stirred vigorously for 10 minutes. The mixture was diluted with $CHCl_3$, washed with sat. aq. $NaHCO_3$, H_2O and brine, dried over Na_2SO_4, filtered and concentrated *in vacuo*. The product was purified by silica gel column chromatography (pentane/EtOAc, 50:1→20:1) affording compound **24** (197 mg, 40% over two steps) as a white solid. ^{1}H NMR (850 MHz, $CDCl_3$) δ 7.86 – 7.78 (m, 6H, 6CH Ar), 7.76 (dd, *J* = 8.1, 1.4 Hz, 1H, CH Ar), 7.74 – 7.70 (m, 3H, 3CH Ar), 7.68 (d, *J* = 1.7 Hz, 1H, CH Ar), 7.63 (dd, *J* = 7.9, 1.3 Hz, 1H, CH Ar), 7.58 (dd, *J* = 8.4, 1.7 Hz, 1H, CH Ar), 7.51 – 7.39 (m, 8H, 8CH Ar), 5.19 – 5.16 (m, 2H, CH_2 Nap), 4.98 (d, *J* = 10.7 Hz, 1H, CH*H* Nap), 4.93 (d, *J* = 11.3 Hz, 1H, CH*H* Nap), 4.85 (t, *J* = 3.4 Hz, 1H, H2), 4.80 (dd, *J* = 11.3, 4.2 Hz, 2H, CH_2 Nap), 4.66 (dd, *J* = 11.3, 1.4 Hz, 1H, H6a), 4.27 (dd, *J* = 11.2, 2.9 Hz, 1H, H6b), 4.10 – 4.06 (m, 2H, H4 and H1), 3.48 (dd, *J* = 11.2, 9.2 Hz, 1H, H7), 2.84 (dd, *J* = 9.3, 3.8 Hz, 1H, H3), 2.76 (dddd, *J* = 11.2, 4.4, 2.9, 1.4 Hz, 1H, H5). ^{13}C NMR (214 MHz, $CDCl_3$) δ 153.5 (C=N), 135.9, 135.4, 135.0, 133.4, 133.4, 133.4, 133.3, 133.2, 133.1 (9C_q Ar), 128.5, 128.5, 128.3, 128.2, 128.1, 128.1, 127.9, 127.8, 127.8, 127.5, 127.2, 127.1, 126.5, 126.5, 126.3, 126.3, 126.2, 126.2, 126.2, 126.0, 126.0 (21CH Ar), 91.4 (CCl_3), 85.2 (C4), 77.2 (C3), 76.4, 76.2 (2CH_2 Nap), 76.1 (C7), 72.4 (CH_2 Nap), 68.3 (C6), 58.7 (C1), 36.3 (C2), 33.8 (C5). HRMS (ESI) m/z: [M+H]$^+$ calc for $C_{42}H_{36}Cl_3INO_4$ 850.07491, found 850.07431.

((1*R*,2*R*,3*R*,4*S*,5*S*,6*R*)-3,4,5-Tris(naphthalen-2-ylmethoxy)-7-octyl-7-azabicyclo[4.1.0]heptan-2-yl)methanol (**25**)

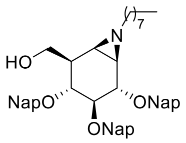

Compound **24** (190 mg, 0.22 mmol) was dissolved in a mixture of DCM/MeOH (1/1, 1 mL/1 mL) and HCl (1.25 M in MeOH, 0.53 mL, 0.66 mmol) was added. The mixture was stirred overnight and subsequently neutralized by addition of Amberlite IRA-67. After stirring at rt for 20 h, the reaction mixture was filtered and the resin was washed with DCM/MeOH (3 x). The filtrate was concentrated to afford an oil which was co-evaporated with toluene (3 x) and directly taken up in dry DMF (2 mL). Then K_2CO_3 (39 mg, 0.28 mmol) and 1-iodooctane (86 µL, 0.48 mmol) were added. The mixture was heated up to 80 °C and stirred for 16 h. After cooling to rt, the mixture was diluted with H_2O, extracted with EtOAc (2 x), the combined organic layers were washed with water (2 x) and brine, dried over Na_2SO_4, filtered and concentrated *in vacuo*. The product was purified by silica gel column chromatography (pentane/EtOAc, 7:1→4:1) affording compound **25** (55 mg, 35% over two steps) as a pale yellow solid. ^{1}H NMR (500 MHz, $CDCl_3$) δ 7.83 – 7.69 (m, 9H, 9CH Ar), 7.69 – 7.60 (m, 3H, 3CH Ar), 7.52 – 7.35 (m, 9H, 9CH Ar), 5.15 – 4.85 (m, 5H, 5CH*H* Nap), 4.74 (d, *J* = 10.9 Hz, 1H, 1CH*H* Nap), 4.03 – 3.90 (m, 2H, H6ab), 3.90 – 3.82 (m, 1H, H2), 3.66 – 3.57 (m, 2H, H3 and H4), 2.95 – 2.80 (br s, 1H, OH), 2.23 (dt, *J* = 11.5, 7.2 Hz, 1H, NCH*H*), 2.17 – 2.06 (m, 2H, H5 and NCH*H*), 1.84 (dd, *J* = 6.3, 3.5 Hz, 1H, H7), 1.60 (d, *J* = 6.3 Hz, 1H, H1), 1.46 (p, *J* = 7.7 Hz, 2H, CH_2), 1.32 – 1.17 (m, 10H, 5CH_2), 0.89 (t, *J* = 7.0 Hz, 3H, CH_3). ^{13}C NMR (126 MHz, $CDCl_3$) δ 136.6, 136.1, 135.7, 133.5, 133.4, 133.4, 133.2, 133.1, 133.0 (9C_q Ar), 128.4, 128.2, 128.1, 128.1, 127.8, 127.8, 127.7, 126.9, 126.7, 126.4, 126.3, 126.1, 126.1, 126.1, 126.1, 125.9, 125.8 (21CH Ar), 85.6 (C3), 81.5 (C2), 77.1 (C4), 75.8, 75.4, 73.0 (3CH_2 Nap), 63.9 (C6), 61.1 (NCH_2), 43.3 (C5), 42.6 (C7), 40.7 (C1), 32.0, 29.6, 29.6, 29.4, 27.5, 22.8 (6CH_2), 14.3 (CH_3). HRMS (ESI) m/z: [M+H]$^+$ calc for $C_{48}H_{54}NO_4$ 708.40474, found 708.40436.

(1*R*,2*S*,3*R*,4*S*,5*S*,6*R*)-2-(Azidomethyl)-3,4,5-tris(naphthalen-2-ylmethoxy)-7-octyl-7-azabicyclo[4.1.0]heptane (**26**)

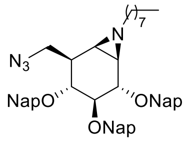

Compound **25** (50 mg, 0.07 mmol) was dissolved in dry DCM (0.7 mL) in a microwave tube. Then Et_3N (19 µL, 0.14 mmol) and *N*-methyl imidazole (40 µL, 0.5 mmol) were added and the mixture was cooled to 0 °C. Tosylchloride (40 mg, 0.21 mmol) was added at 0 °C. The tube was then sealed and the mixture was stirred at rt for 28 h. The reaction was quenched with H_2O at 0 °C, diluted with EtOAc, washed with diluted aq. 1M HCl, sat. aq. $NaHCO_3$, H_2O and brine, dried over Na_2SO_4, filtered and concentrated *in vacuo*. After co-evaporation with toluene (2 x), the crude intermediate was dissolved in dry DMF (0.7 mL). NaN_3 (45 mg, 0.7 mmol) was added and the mixture was heated up to 50 °C and stirred for 40 h. After cooling to rt, the mixture was diluted with H_2O, extracted with EtOAc (2 x), the combined organic layers were washed with H_2O (2 x) and brine, dried over Na_2SO_4, filtered and concentrated *in vacuo*. The product was purified by silica gel column chromatography (pentane/EtOAc, 18:1→10:1) affording compound **26** (35 mg, 68% over two steps) as a white solid. ^{1}H NMR (400 MHz, $CDCl_3$) δ 7.84 – 7.58 (m, 12H, 12CH Ar), 7.53 – 7.31 (m, 9H, 9CH Ar), 5.10 – 4.85 (m, 5H, 5CH*H* Nap), 4.60 (d, *J* = 11.2 Hz, 1H, 1CH*H* Nap), 3.89 (d, *J* = 8.2 Hz, 1H, H2), 3.76 (dd, *J* = 11.6, 3.5 Hz, 1H, H6a), 3.56 (dd, *J* = 10.0, 8.1 Hz, 1H, H3), 3.31 (dd, *J* = 11.6, 10.0 Hz, 1H, H6b), 3.23 (t, *J* = 10.0 Hz, 1H, H4), 2.22 – 2.09 (m, 3H, H5 and NCH_2), 1.88 (dd, *J* = 6.1, 3.1 Hz, 1H, H7), 1.70 (d, *J* = 6.1 Hz, 1H, H1), 1.44 (tt, *J* = 9.2, 6.9, 3.1 Hz, 2H, CH_2), 1.26 (d, *J* = 6.3 Hz, 10H, 5CH_2), 0.89 (t, *J* = 6.7 Hz, 3H, CH_3). ^{13}C NMR (101 MHz, $CDCl_3$) δ 136.5, 135.9, 135.6, 133.4, 133.4, 133.4, 133.2, 133.1, 133.0 (9C_q Ar), 128.4, 128.3, 128.1, 128.1, 128.0, 127.8, 127.8, 126.9, 126.7, 126.4, 126.3, 126.2, 126.1, 126.1, 126.1, 126.0, 126.0, 125.8 (21CH Ar), 85.7 (C3), 81.5 (C2), 77.3 (C4), 75.6, 75.4, 72.9 (3CH_2 Nap), 61.1 (NCH_2), 52.3 (C6), 42.3 (C5), 42.1 (C7), 42.0 (C1), 32.0, 29.6, 29.6, 29.4, 27.5, 22.8 (6CH_2), 14.4 (CH_3). HRMS (ESI) m/z: [M+H]$^+$ calc for $C_{48}H_{53}N_4O_3$ 733.41122, found 733.41092.

(1*R*,2*S*,3*S*,4*R*,5*S*,6*R*)-5-(Azidomethyl)-7-octyl-7azabicyclo[4.1.0]heptane-2,3,4-triol (**27**)

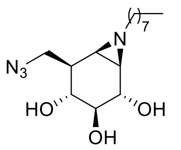

Compound **26** (31 mg, 42 µmol) was dissolved in a mixture of DCM/H_2O (10/1, 0.8 mL/0.08 mL) and DDQ (39 mg, 0.17 mmol) was added. Then the mixture was stirred at rt for 24 h until full conversion was observed by LC-MS. The reaction was quenched with a mixture of sat. aq. $NaHCO_3$ and sat. aq. $Na_2S_2O_3$, extracted with EtOAc (3 x), the combined organic layers were washed with brine, dried over Na_2SO_4, filtered and concentrated *in vacuo*. The product was purified by silica gel column chromatography (DCM/MeOH, 100:1→19:1) and by semi-preparative reversed phase HPLC (linear gradient. Solution used: A: 50 mM NH_4HCO_3 in H_2O, B: MeCN) affording compound **27** (8.71 mg, 66%) as a white powder after lyophilization. ^1H NMR (850 MHz, MeOD) δ 3.83 (dd, *J* = 11.8, 3.6 Hz, 1H, H6a), 3.58 (d, *J* = 8.3 Hz, 1H, H2), 3.34 – 3.31 (m, 1H, H6b), 3.09 (dd, *J* = 10.0, 8.3 Hz, 1H, H3), 2.98 (t, *J* = 9.8 Hz, 1H, H4), 2.41 (ddd, *J* = 11.7, 8.9, 6.5 Hz, 1H, NCH*H*), 2.11 (ddd, *J* = 11.8, 8.8, 5.7 Hz, 1H, NCH*H*), 1.95 – 1.92 (m, 1H, H7), 1.90 (dt, *J* = 9.9, 3.5 Hz, 1H, H5), 1.67 (d, *J* = 6.2 Hz, 1H, H1), 1.60 – 1.53 (m, 2H, CH_2), 1.41 – 1.27 (m, 10H, 5CH_2), 0.91 (t, *J* = 7.2 Hz, 3H, CH_3). ^{13}C NMR (214 MHz, MeOD) δ 78.9 (C3), 73.9 (C2), 69.9 (C4), 62.1 (NCH_2), 53.5 (C6), 45.8 (C1), 43.6 (C5), 43.1 (C7), 33.0, 30.7, 30.5, 30.4, 28.4, 23.7 (6CH_2), 14.4 (CH_3). HRMS (ESI) m/z: $[M+H]^+$ calc for $C_{15}H_{29}N_4O_3$ 313.22342, found 313.22312.

3,3-Dimethyl-1-(6-oxo-6-(((1-(((1*R*,2*S*,3*R*,4*S*,5*S*,6*R*)-3,4,5-trihydroxy-7-octyl-7-azabicyclo[4.1.0]heptan-2-yl)methyl)-1*H*-1,2,3-triazol-4-yl)methyl)amino)hexyl)-2-((1*E*,3*E*)-5-((*E*)-1,3,3-trimethylindolin-2-ylidene)penta-1,3-dien-1-yl)-3*H*-indol-1-ium (**ABP 6**)

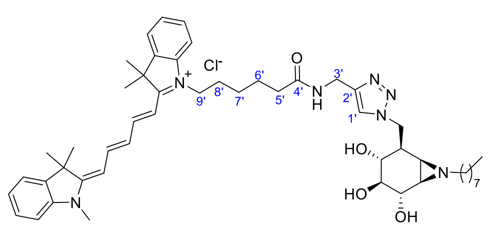

Compound **27** (3.97 mg, 12.7 µmol) was dissolved in DMF (0.5 mL). Then Cy5-alkyne (10.6 mg, 19 µmol), $CuSO_4$ (0.4 mg, 2.54 µmol) and sodium ascorbate (1 mg, 5.1 µmol) were added and the mixture was stirred overnight at rt until full conversion was observed by LC-MS. The solvent was evaporated and the product was purified by semi-preparative reversed phase HPLC (linear gradient. Solution used: A: 50 mM NH_4HCO_3 in H_2O, B: MeCN) affording ABP **6** (3.33 mg, 30%) as a blue powder after lyophilization. ^1H NMR (850 MHz, MeOD) δ 8.24 (td, *J* = 13.0, 3.9 Hz, 2H, 2CH=CH), 7.89 (s, 1H, H1'), 7.49 (dd, *J* = 7.4, 1.2 Hz, 2H, 2CH Ar), 7.41 (qd, *J* = 7.5, 7.1, 1.2 Hz, 2H, 2CH Ar), 7.32 – 7.24 (m, 4H, 4CH Ar), 6.61 (t, *J* = 12.3 Hz, 1H, CH=CH), 6.28 (d, *J* = 13.6 Hz, 2H, 2CH=CH), 4.74 (dd, *J* = 13.5, 3.6 Hz, 1H, H6a), 4.51 (dd, *J* = 13.5, 10.0 Hz, 1H, H6b), 4.43 (s, 2H, H3'ab), 4.09 (t, *J* = 7.6 Hz, 2H, H9'ab), 3.63 (s, 3H, NCH_3 Cy5), 3.59 (d, *J* = 7.8 Hz, 1H, H2), 3.15 – 3.08 (m, 2H, H4 and H3), 2.51 (ddd, *J* = 11.7, 8.4, 6.3 Hz, 1H, NCH*H* octyl), 2.28 – 2.21 (m, 3H, H5 and H5'ab), 1.87 – 1.80 (m, 3H, NCH*H* octyl and H8'ab), 1.74 – 1.68 (m, 14H, H6'ab and 4CH_3 Cy5), 1.57 – 1.43 (m, 6H, H1, H7, H7'ab and CH_2 octyl), 1.35 – 1.23 (m, 10H, 5CH_2 octyl), 0.88 (t, *J* = 7.1 Hz, 3H, CH_3 octyl). ^{13}C NMR (214 MHz, MeOD) δ 175.7, 175.5, 174.6 (C4' and C=N and *C*=CH), 155.6, 155.5 (2CH=CH), 146.4 (C2'), 144.3, 143.6, 142.6, 142.5 (4C_q Ar), 129.8, 129.7, 126.6, 126.3, 126.2 (CH Ar and CH=CH), 124.6 (C1'), 123.4, 123.3, 112.0, 111.9, 104.4, 104.2 (CH Ar and CH=CH), 79.0 (C3), 74.0 (C2), 70.1 (C4), 62.1 (NCH_2 octyl), 52.0 (C6), 50.5 (C_q), 50.4 (C_q), 45.8 (C1), 44.9 (C5), 44.8 (C9'), 42.4 (C7), 36.5 (C5'), 35.7 (C3'), 33.0 (CH_2 octyl), 31.5 (NCH_3 Cy5), 30.7, 30.6, 30.4, 28.5 (4CH_2 octyl), 28.1 (C8'), 28.0 (2CH_3 Cy5), 27.8 (2CH_3 Cy5), 27.3 (C7'), 26.4 (C6'), 23.7 (CH_2 octyl), 14.5 (CH_3 octyl). HRMS (ESI) m/z: $[M]^+$ calc for $C_{50}H_{70}N_7O_4$ 832.54838, found 832.54775.

Synthesis of ABP 7

6-(Trityloxy)hexan-1-ol (**10**)

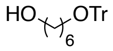

Tritylchloride (2.78 g, 10 mmol) was added to a solution of 1,6-hexanediol (11.8 g, 100 mmol) and pyridine (1.6 mL, 20 mmol) in DCM (100 mL) at room temperature. After vigorously stirring for 90 min, the solution was washed with brine (3x). The organic layer was dried with $MgSO_4$, filtrated and concentrated *in vacuo*. Purification by column chromatography (30% EtOAc in pentane) gave tritylhexanol **10** (3.41 g, 9.47 mmol, 95%) as a white solid. Spectroscopic data are in agreement with those previously reported[1,2].

(((6-Iodohexyl)oxy)methanetriyl)tribenzene (**11**)

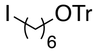

Tritylhexanol **10** (3.35 g, 9.3 mmol) was dissolved in Et_2O (35 mL) and CH_3CN (11 mL). After addition of imidazole (1.90 g, 27.9 mmol) and triphenylphosphine (14.0 mmol, 3.66 g) the reaction mixture was cooled to 0 ºC and iodine (3.54 g, 14.0 mmol) was added in small portions. After stirring for 30 min, the reaction was allowed to warm to room temperature and stirred overnight. The reaction mixture was diluted with Et_2O and the precipitated was filtered off. The filtrated was washed with $Na_2S_2O_3$, dried with $MgSO_4$, filtrated and concentrated *in vacuo*. Purification by column chromatography (pentane → 3% EtOAc in pentane) yielded title compound **11** (3.51 g, 7.46 mmol, 80%) as a clear oil. Spectroscopic data are in agreement with those previously reported[3]

(((6-Azidohexyl)oxy)methanetriyl)tribenzene (**12**)

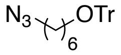

NaN_3 (1.30 g, 20 mmol) was added to a solution of compound **17** (0.92 g, 2.0 mmol) in DMF (5 mL) and stirred at 80 ºC overnight. The reaction mixture was diluted with EtOAc and washed with H_2O (4x). The organic layer was dried with $MgSO_4$, filtrated and concentrated *in vacuo* to obtain the azide **18** (0.787 g, 2.0 mmol) as a clear oil, which was used without further purification. ^1H NMR (400 MHz, $CDCl_3$) δ 7.43 (d, *J* = 7.2 Hz, 6H), 7.32 – 7.16 (m, 9H), 3.21 (t, *J* = 7.0 Hz, 2H), 3.05 (t, *J* = 6.5 Hz, 2H), 1.65 – 1.50 (m, 4H), 1.45 – 1.24 (m, 4H). ^{13}C NMR (101 MHz, $CDCl_3$) δ 144.5, 128.8, 127.8, 126.9, 86.4, 63.5, 51.5, 30.0, 28.9, 26.7, 26.0.

6-(Trityloxy)hexan-1-amine (**13**)

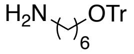

PPh_3 on beads (200 mg, 0.60 mmol, 3 mmol/g) was added to a solution of azide **18** (193 mg, 0.5 mmol) in THF (2.3 mL) and H_2O (9 µL). After stirring for 48 h at room temperature, the reaction mixture was filtered and concentrated *in vacuo*. The crude product was co-evaporated with toluene (3x) to obtain amine **19** (186 mg, 0.517 mmol) as a clear oil which was used without further purification. ^{1}H NMR (400 MHz, $CDCl_3$) δ 7.44 (d, J = 7.6 Hz, 6H), 7.31 – 7.18 (m, 9H), 3.05 (t, J = 6.6 Hz, 2H), 2.63 (t, J = 7.0 Hz, 2H), 1.68 – 1.54 (m, 2H), 1.43 – 1.31 (m, 4H), 1.31 – 1.22 (m, 2H), 1.21 (s, 1H). ^{13}C NMR (101 MHz, CDCl3) δ 144.5, 128.7, 127.7, 126.9, 86.3, 63.6, 42.3, 33.8, 30.1, 26.8, 26.2. HRMS: Calculated for $[C_{25}H_{30}NO]^+$ 360.23219, found 360.23228.

(1*R*,2*R*,4*R*)-*N*-(6-(Trityloxy)hexyl)bicyclo[2.2.1]hept-5-ene-2-carboxamide (exo, **15**) and (1*R*,2*S*,4*R*)-*N*-(6-(trityloxy)hexyl)bicyclo[2.2.1]hept-5-ene-2-carboxamide (endo, **16**)

To a solution of amine **13** (0.90 g, 2.52 mmol) in DCE (12.6 mL) norbornene-OSu **14**[4] (0.61 g, 2.59 mmol; mixture of *endo* and *exo,* mostly *endo*) and DIPEA (1.07 mL) was added at room temperature. After stirring overnight the reaction mixture was concentrated *in vacuo*. Purification by column chromatography (20% EtOAc in pentane → 30% EtOAc in pentane) gave *exo*-product **15** (0.34 g, 0.70 mmol, 28%) and *endo*-product **16** (0.82 g, 1.7 mmol, 68%) as a clear oil.

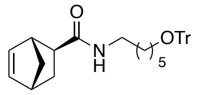

Exo-product **15** ^{1}H NMR (400 MHz, $CDCl_3$) δ 7.47 – 7.40 (m, 6H), 7.30 – 7.20 (m, 9H), 6.13 (dd, J = 5.5, 3.0 Hz, 1H), 6.08 (dd, J = 5.4, 3.2 Hz, 1H), 5.51 (s, 1H), 3.23 (d, J = 6.3 Hz, 1H), 3.20 (d, J = 6.5 Hz, 1H), 3.06 (d, J = 6.3 Hz, 1H), 3.03 (d, J = 6.5 Hz, 1H), 2.90 – 2.87 (m, 2H), 1.96 – 1.86 (m, 2H), 1.72 (d, J = 8.3 Hz, 1H), 1.66 – 1.57 (m, 2H), 1.52 – 1.44 (m, 2H), 1.41 – 1.34 (m, 2H), 1.33 – 1.31 (m, 1H), 1.31 – 1.25 (m, 3H). ^{13}C NMR (101 MHz, $CDCl_3$) δ 175.6, 144.5, 138.3, 136.1, 128.8, 127.8, 126.9, 86.4, 63.5, 47.3, 46.5, 44.8, 41.7, 39.7, 30.6, 30.0, 29.8, 26.9, 26.1. HRMS: Calculated for $[C_{33}H_{37}NO_2Na]^+$ 502.27165, found 502.27059.

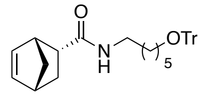

Endo-product **16** ^{1}H NMR (400 MHz, $CDCl_3$) δ 7.51 – 7.39 (m, 6H), 7.32 – 7.19 (m, 9H), 6.21 (dd, J = 5.6, 3.1 Hz, 1H), 5.95 (dd, J = 5.6, 2.8 Hz, 1H), 5.39 (s, 1H), 3.17 (dd, J = 7.1, 2.6 Hz, 1H), 3.14 (dd, J = 7.2, 2.5 Hz, 1H), 3.10 (s, 1H), 3.04 (t, J = 6.6 Hz, 2H), 2.89 (s, 1H), 2.85 – 2.80 (m, 1H), 1.94 – 1.87 (ddd, J = 11.9, 9.4, 3.8 Hz, 1H), 1.65 – 1.55 (m, 2H), 1.46 – 1 .40 (m, 3H), 1.40 – 1.34 (m, 2H), 1.34 – 1.30 (m, 1H), 1.30 – 1.22 (m, 3H). ^{13}C NMR (101 MHz, $CDCl_3$) δ 174.2, 144.5, 137.8, 132.4, 128.7, 127.8, 126.9, 86.4, 63.5, 50.1, 46.3, 44.9, 42.8, 39.5, 30.0, 29.7, 26.9, 26.1. *Endo*-product **19** HRMS: Calculated for $[C_{33}H_{37}NO_2Na]^+$ 502.27165, found 502.27141.

(1R,2R,4R)-*N*-(6-Hydroxyhexyl)bicyclo[2.2.1]hept-5-ene-2-carboxamide (**17**)

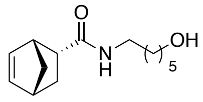

Compound **16** (0.815 g, 1.7 mmol) was dissolved in a mixture of DCM/MeOH (20 mL, 1:1) and *p*-toluenesulfonic acid was added until pH 2. After stirring overnight at room temperature, the reaction mixture was neutralized with a saturated aqueous $NaHCO_3$ solution. The aqueous layer was extracted with EtOAc (3x) and the combined organic layers were washed with brine, dried with MgSO4, filtrated and concentrated *in vacuo*. Purification by column chromatography (5% Methanol in DCM → 8% Methanol in DCM) gave title compound **17** (0.349 g, 1.47 mmol, 86%) as a clear yellow oil. ^{1}H NMR (400 MHz, $CDCl_3$) δ 6.22 (dd, J = 5.6, 3.1 Hz, 1H), 5.96 (dd, J = 5.6, 2.8 Hz, 1H), 5.80 (s, 1H), 3.60 (t, J = 6.5 Hz, 1H), 3.23 – 3.15 (m, 2H), 3.13 (s, 1H), 2.91 (s, 1H), 2.89 – 2.80 (m, 2H), 1.92 (ddd, J = 11.8, 9.4, 3.7 Hz, 1H), 1.59 – 1.51 (m, 2H), 1.50 – 1.41 (m, 3H), 1.40 – 1.22 (m, 6H). ^{13}C NMR (101 MHz, $CDCl_3$) δ 174.4, 137.7, 132.2, 62.3, 50.0, 46.2, 44.7, 42.7, 39.2, 32.5, 29.8, 29.6, 26.5, 25.3. HRMS: Calculated for $[C_{14}H_{24}NO_2]^+$ 238.18016, found 238.18026.

(1R,2R,4R)-*N*-(6-Iodohexyl)bicyclo[2.2.1]hept-5-ene-2-carboxamide (**18**)

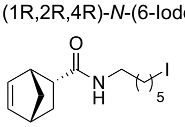

To a solution of alcohol **17** (119 mg, 0.50 mmol) in THF (2.5 mL) was added PPh_3 (197 mg, 0.75 mmol) and imidazole (64 mg, 1.0 mmol). The reaction mixture was stirred under reflux. Carefully a solution of I_2 (190 mg, 0.75 mmol) in THF (1.0 mL) was added. After stirring for 1.5 h, the reaction mixture was concentrated, redissolved in EtOAc and washed with $Na_2S_2O_3$. The organic layer was dried with $MgSO_4$, filtrated, and concentrated *in vacuo*. Purification by column chromatography (30% EtOAc in pentane) yielded title compound **18** (126 mg, 0.361 mmol, 73%) as a clear yellow oil. ^{1}H NMR (400 MHz, $CDCl_3$) δ 6.24 (dd, J = 5.6, 3.1 Hz, 1H), 5.97 (dd, J = 5.6, 2.8 Hz, 1H), 5.39 (s, 1H), 3.25 – 3.15 (m, 4H), 3.13 (s, 1H), 2.92 (s, 1H), 2.86 (dt, J = 9.2, 4.0 Hz, 1H), 1.94 (ddd, J = 11.9, 9.4, 3.8 Hz, 1H), 1.83 (t, J = 7.0 Hz, 1H), 1.80 (t, J = 7.0 Hz, 1H), 1.54 – 1.36 (m, 5H), 1.35 – 1.27 (m, 4H). ^{13}C NMR (101 MHz, $CDCl_3$) δ 174.2, 137.7, 132.2, 50.0, 46.2, 44.7, 42.7, 39.2, 33.3, 30.1, 29.8, 29.5, 25.8, 7.2. HRMS: Calculated for $[C_{14}H_{23}INO]^+$ 348.08188, found 348.08177.

(1*S*,2*S*,4*S*)-*N*-(6-((1*R*,2*S*,3*S*,4*S*,5*R*,6*R*)-2,3,4-Trihydroxy-5-(hydroxymethyl)-7-azabicyclo[4.1.0]heptan-7-yl)hexyl)bicyclo[2.2.1]hept-5-ene-2-carboxamide (**19**)

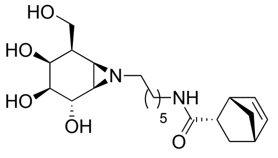

A solution of norbornene **18** (83 mg, 0.24 mmol) in DMF (2 mL) was added to aziridine **4** (0.2 mmol). K_2CO_3 (119 mg, 0.86 mmol) was added to the reaction mixture followed by heating to 75 ºC. After stirring overnight, the reaction mixture was concentrated *in vacuo*. Purification by column chromatography (12% MeOH in DCM) followed by HPLC purification (A: 50 mM NH_4HCO_3 in H_2O; B: MeCN; 20% → 26%, A in B) and lyophilisation resulted in compound **19** (24.3 µmol, 9.6 mg, 12%). LC/MS analysis: R_t 3.81 min (linear gradient 10→90% B in 12.5 min), m/z 395.27 [M + H]$^+$. ^{1}H NMR (400 MHz, D_2O) δ 6.23 (dd, J = 5.6, 3.1 Hz, 1H), 5.90 (dd, J = 5.6, 2.8 Hz, 1H), 3.94 (d, J = 8.2 Hz, 1H), 3.79 (d, J = 7.4 Hz, 2H), 3.75 (s, 1H), 3.33 (dd, J = 8.2, 2.2 Hz, 1H), 3.16 – 3.05 (m, 3H), 2.97 – 2.92 (m, 1H), 2.90 (s, 1H), 2.50 – 2.42 (m, 1H), 2.18 – 2.04 (m, 2H), 2.02 (d, J = 6.1 Hz, 1H), 1.86 – 1.82 (m,

2H), 1.52 – 1.40 (m, 4H), 1.39 – 1.22 (m, 7H). ^{13}C NMR (101 MHz, D_2O) δ 177.7, 138.4, 131.8, 76.2, 70.6, 70.4, 61.1, 58.4, 49.5, 46.4, 44.1, 42.6, 42.5, 40.4, 39.2, 39.1, 29.5, 28.7, 28.3, 26.1, 25.7. HRMS: Calculated for $[C_{21}H_{35}N_2O_5]^+$ 395.25405, found 395.25336.

ABP 7

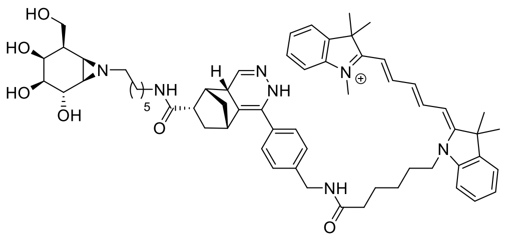

Cy5 tetrazine **20** (2.83 mg, 4.3 µmol) was added to a solution of norbornene **19** (0.94 mg, 2.38 µmol) in MeOH (4 mL). After stirring overnight at rt, the reaction mixture was concentrated in vacuo and purified by HPLC under neutral conditions (A: 50 mM NH_4HCO_3 in H2O; B: MeCN; 49% → 52%, A in B) yielding ABP **7** (2.12 mg, 2.07 µmol, 87%) as a mixture of regioisomers. HRMS: Calculated for $[C_{62}H_{80}N_7O_6]^+$ 1018.61646, found 1018.61611.

Biochemical Methods: General Experimental Details

Recombinant human GBA (rhGBA, imiglucerase, Cerezyme®) and GAA (rhGAA, alglucosidase alfa, Myozyme) were obtained from Sanofi Genzyme (Cambridge, MA, USA). HEK293T (CRL-3216) cell lines were purchased from ATCC (Manassas, VA, USA). Cell lines were cultured in DMEM medium (Sigma-Aldrich, St. Lois, MO, USA), supplied with 10% (v/v) FCS, 0.1% (w/v) penicillin/streptomycin and 1% (v/v) Glutamax, under 5% CO_2 at 37°C. The generation of HEK293T cells overexpressing GBA2[5] and the preparation of cellular homogenates were performed as previously described[6]. Mouse tissue were isolated according to guidelines approved by the ethical committee of Leiden University (DEC#13191). All the tissue lysates were prepared in potassium phosphate lysis buffer (25 mM in pH 6.5, supplemented with 0.1% (v/v) Triton X-100 and protease inhibitor 1x cocktail (Roche)) via homogenization with silent crusher S equipped with Typ 7 F/S head (30 rpm x 1000, 3 × 7 sec) on ice and lysate concentration was determined with Bicinchoninic acid (BCA) Protein Assay Kit (PierceTM)[7]. The protein fractions were stored in small aliquots at -80 °C until use.

***In situ* Labeling of HEK 293T cells and SDS-PAGE analysis**

Experiment was conducted based on the previously described methods[5] The HEK293T cells which contain endogenous GBA and overexpressed GBA2 were cultured in 6-well plates and let them grow to at least 80% confluency before experiment. ABP **6**, ABP **8** and ABP **9** were diluted with DMSO into various concentrations (200x of the final concentration), and 5 µL of ABP at different concentrations were added into 1 mL fresh medium containing cells and incubated at 37°C for 24 h. After incubation, cells were washed 3 times with PBS, detached from culture dishes by scraping and lysed in 90 µL of 25 mM KPi buffer (pH 6.5 supplemented with 0.1% (v/v) Triton X-100 and protease inhibitor cocktail) by sonicator. The protein concentrations were determined using BCA kit. For SDS-PAGE, each sample containing the same amount of protein (25 µg total protein) was diluted with 25 mM KPi buffer pH 6.5 (+0.1% (v/v) Triton X-100 and protease inhibitor cocktail) into a total volume of 15 µL, denatured by incubation with 4 µL Laemmli (5x) sample buffer at 98 °C for 5 minutes, and proceeded to SDS-PAGE and fluorescent detection as described above.

pH Dependent Activity-based labelling of rhGBA by ABP 6 and ABP 9

rhGBA produced in an insect-baculovirus expression vector system (BEVS) was diluted to 200 nM in 150 mM McIlvaine buffer pH 4.5, 5.2 or 7.4 (supplemented with 0.1 % (v/v) Triton X-100 and 0.2 % (w/v) sodium taurocholate). ABP 6 or ABP 9 were added to 150 nM in a final reaction volume of 10 µL. The reactions were incubated at 37 °C for 30 mins and stopped by denaturing with Laemmli (x3) sample buffer at 95 °C for 5 minutes. The samples were resolved by electrophoresis in 10% SDS-PAGE gels, running at a constant of 200V for approximately 50 minutes. Wet slab gels were scanned on fluorescence using an Amersham Typhoon 5 Imager (GE Healthcare) with λ_{EX} 635 nm; λ_{EM} > 665 nm.

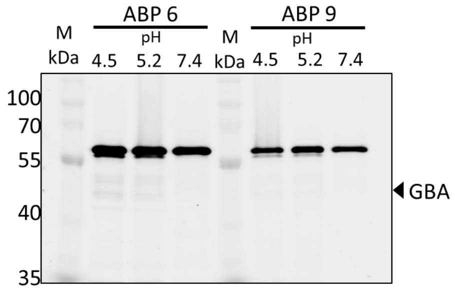

Figure S1: pH dependent labelling of rhGBA (200 nM) with ABP 6 or ABP 9 (150 nM) showing greater labelling at acidin pH (4.5 and 5.2) than neutral pH (pH 7.4)

Crystallisation

Table S1: Data collection and Processing

Compound	1	2	3	4	5	6
PDB Entry	6YTP	6YTR	6YUT	6YV3	6Z39	6Z3I
Diffraction source	Diamond Beamline i02	Diamond Beamline i03	Diamond Beamline i02	Diamond Beamline i02	Diamond Beamline i03	Diamond Beamline i04
Wavelength (Å)	0.979490	0.97630	0.979490	0.979500	0.97630	0.979507
Temperature (K)	100	100	100	100	100	100
Detector	Eiger2 XE 16M	Eiger2 XE 16M	Eiger2 XE 16M	Eiger2 XE 16M	Eiger2 XE 16M	Eiger2 XE 16M
Rotation range per image (°)	0.1	0.1	0.1	0.1	0.1	0.1
Total rotation range (°)	360	360	360	360	360	360
Space group	$C\ 2\ 2\ 2_1$	$P\ 2_1$	$C\ 2\ 2\ 2_1$	$C\ 2\ 2\ 2_1$	$C\ 2\ 2\ 2_1$	$P\ 2_1$
a, *b*, *c* (Å)	108.9, 285.8, 91,6	53.2, 156.8, 68.2	110.7, 285.5, 91.9	110.6, 285.3, 91.8	111.2, 285.6, 91.6	53.1, 76.7, 68.0
α, β, γ (°)	90, 90, 90	90, 102, 90	90, 90, 90	90, 90, 90	90, 90, 90	90, 102, 90
Resolution range (Å)	68.11-1.70	66.71-1.70	47.59-1.76	72.09-1.80	103.60-1.70	88.49-1.80
Total No. of reflections	302601 (14983)	840589 (38616)	275706 (13689)	258518 (12771)	950706 (44363)	348887 (20199)
No. of unique reflections	156420 (7654)	119412 (5867)	143437 (7028)	134018 (6542)	159511 (7828)	49541 (2966)
Completeness (%)	99.9 (99.9)	99.9 (99.7)	99.8 (99.9)	100 (100)	100 (100)	100 (100)
Redundancy	1.9 (2.0)	7.0 (6.6)	1.9 (1.9)	1.9 (2.0)	6.0 (5.7)	7.0 (6.8)
$\langle I/\sigma(I)\rangle$	10.7 (1.1)	6.3 (1.0)	7.6 (1.2)	7.0 (0.9)	13.3 (2.0)	6.7 (0.)
$R_{meas.}$	0.055 (0.123)	0.172 (1.993)	0.064 (0.538)	0.072 (0.893)	0.099 (1.072)	0.224 (2.547)
$CC_{1/2}$	0.999 (0.531)	0.994 (0.547)	0.997 (0.804)	0.998 (0.589)	0.999 (0.760)	0.995 (0.506)
Overall *B* factor from Wilson plot (Å^2)	34	21	33	30	25	27

Table S2: Structure solution and refinement

Compound	1	2	3	4	5	6
PDB Entry	6YTP	6YTR	6YUT	6YV3	6Z39	6Z3I
Resolution range (Å)	68.11-1.70 (1.73-1.70)	66.71-1.70 (1.73-1.70)	47.59-1.76 (1.79-1.76)	72.09-1.80 (1.83-1.80)	103.60-1.70 (1.73-1.70)	88.49-1.80 (1.84-1.80)
Completeness (%)	99.9 (99.9)	99.9 (99.7)	99.8 (99.9)	100 (100)	100 (100)	100 (100)
No. of reflections, working set	156420	119361	143426	134017	159484	49519
No. of reflections test set	7842	5927	7160	6677	7997	2509
Final R_{cryst}	0.21	0.16	0.20	0.19	0.18	0.19
Final R_{free}	0.23	0.21	0.23	0.22	0.20	0.20
No. of non-H atoms						
Protein	7961	7931	7897	7922	7914	3972
Ligand	277	362	314	264	331	193
Water	661	877	735	829	904	340
Total	8899	9172	8946	9015	9149	4505
R.m.s. deviations						
Bonds (Å)	0.014	0.010	0.014	0.007	0.013	0.013
Angles (°)	1.73	1.59	1.75	1.44	1.67	1.70
Average *B* factors (Å^2)						
Protein	33	25	32	30	24	26
Ligand	61	49	60	61	47	48
Water	42	37	41	39	37	36
Ramachandran plot						
Most favoured (%)	95.5	95.2	95.2	94.7	95.4	94.7
Allowed (%)	3.5	3.8	3.7	4.3	3.6	4.2

NMR Spectra

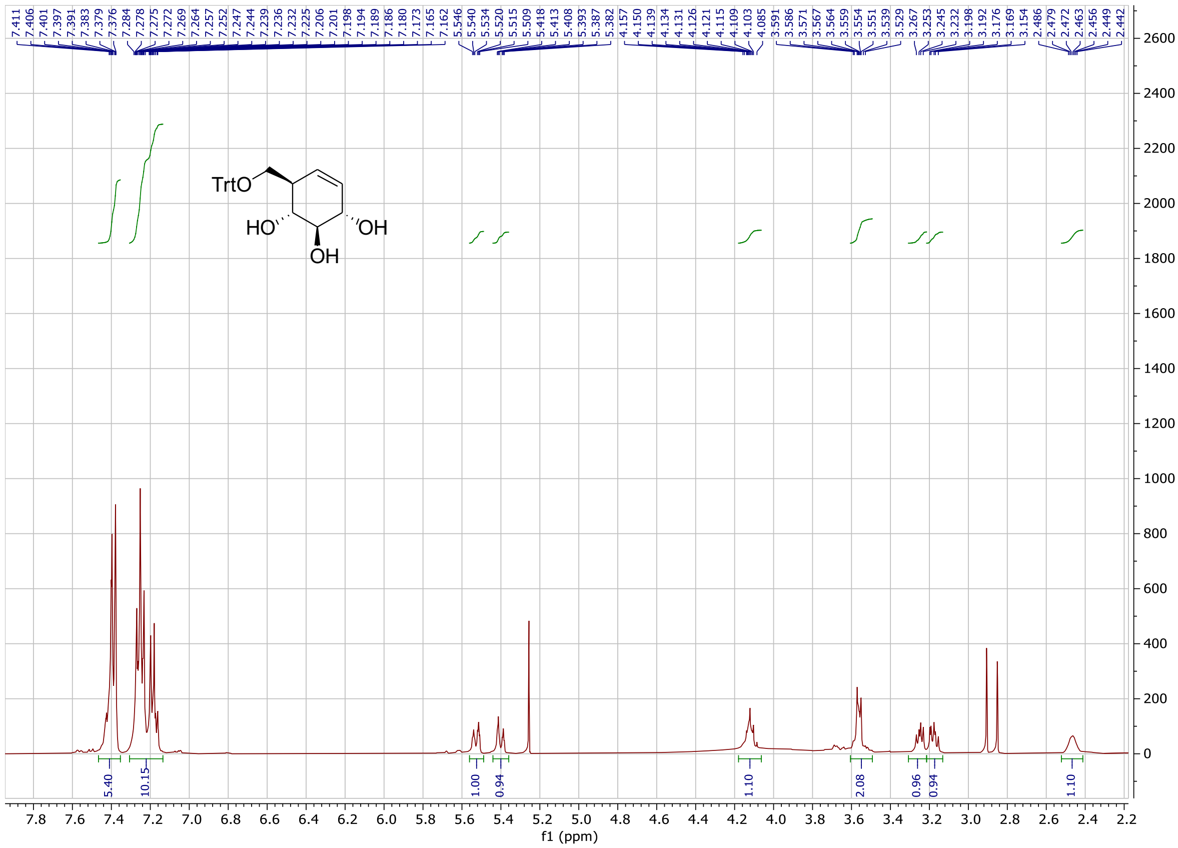

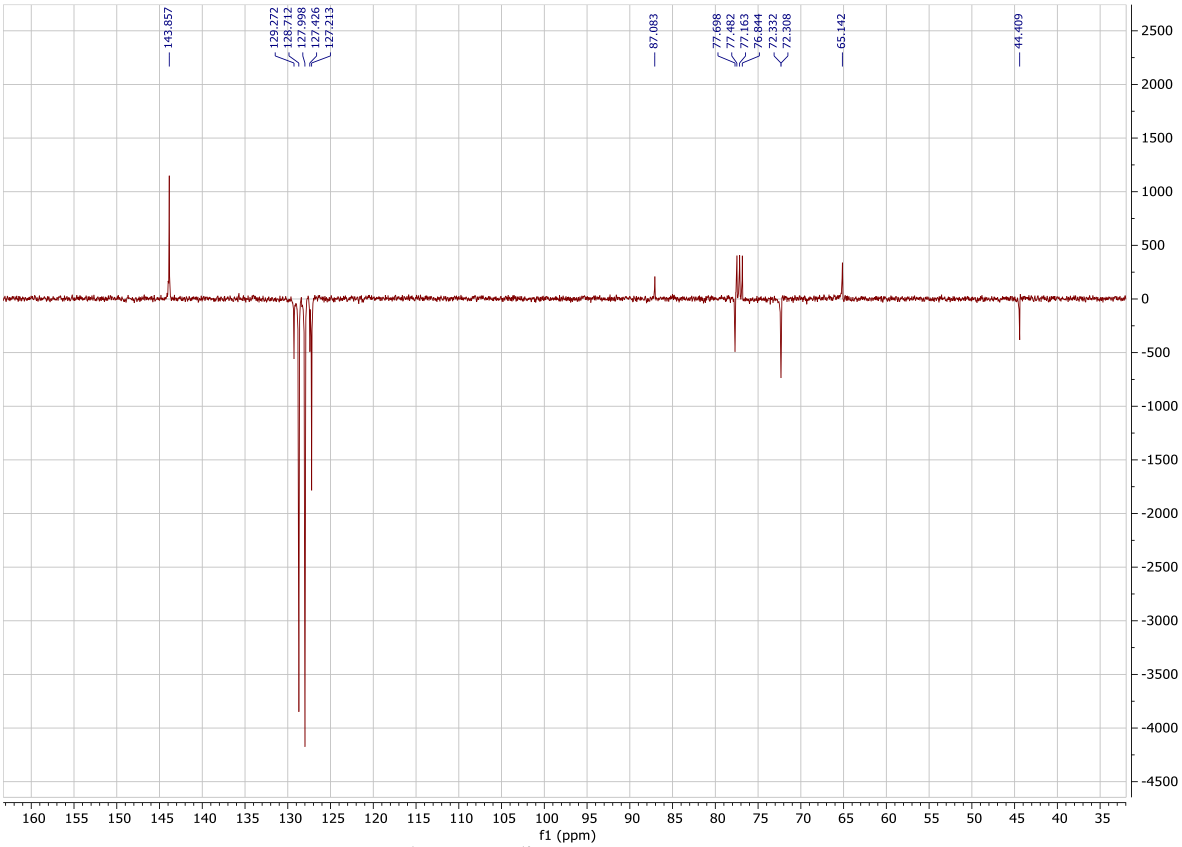

Figure S2: ^{1}H-NMR and ^{13}C-NMR spectra of **22** in $CDCl_3$

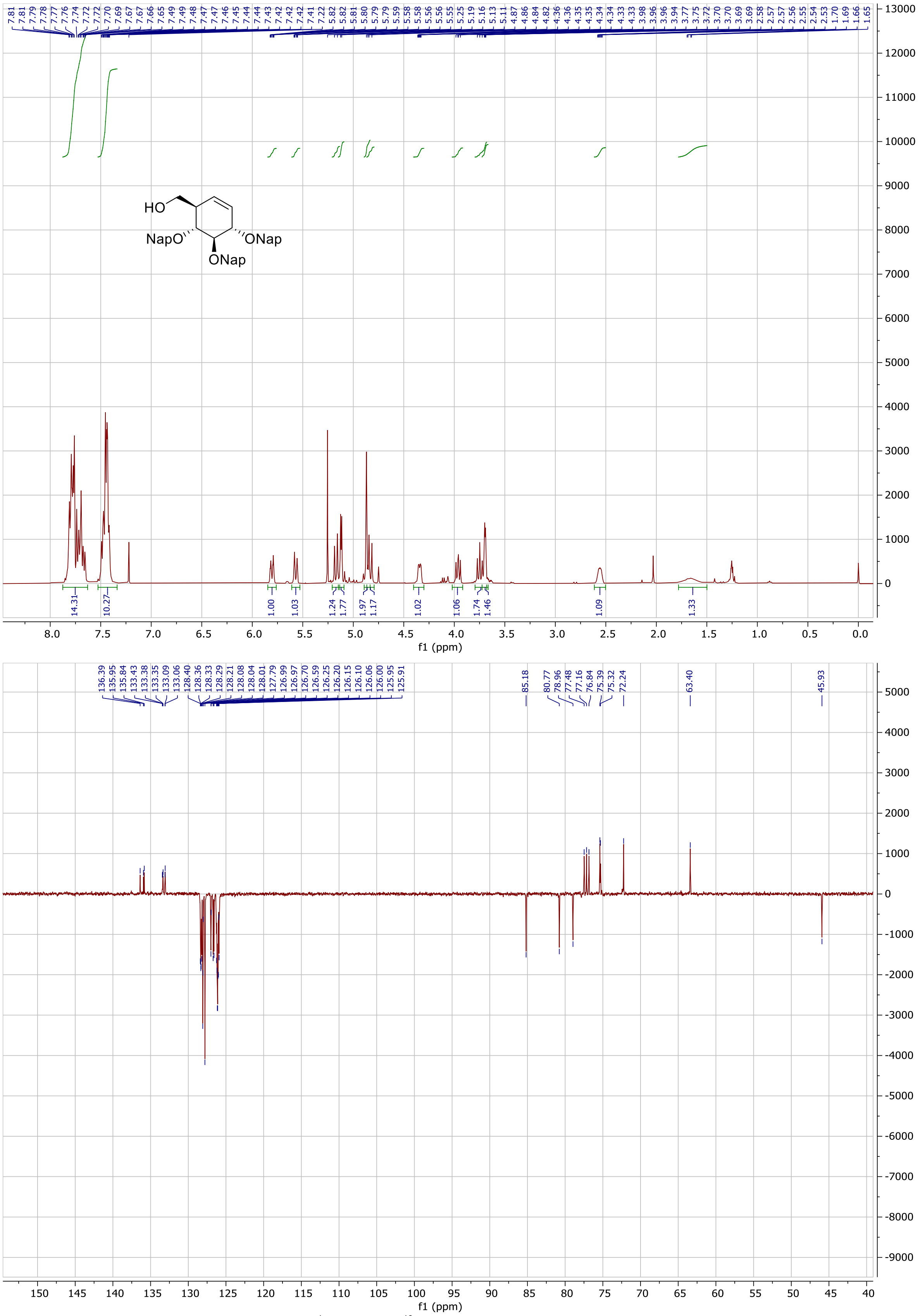

Figure S3: ^{1}H-NMR and ^{13}C-NMR spectra of **23** in $CDCl_3$

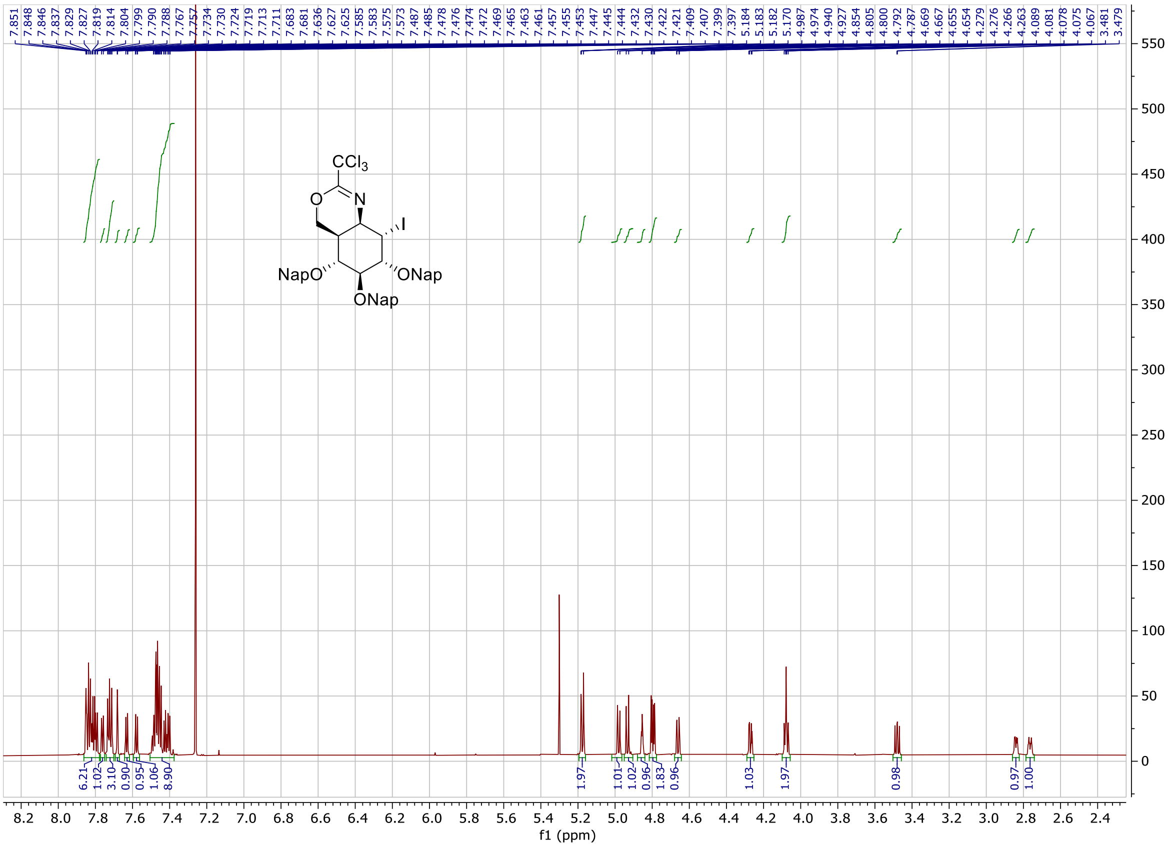

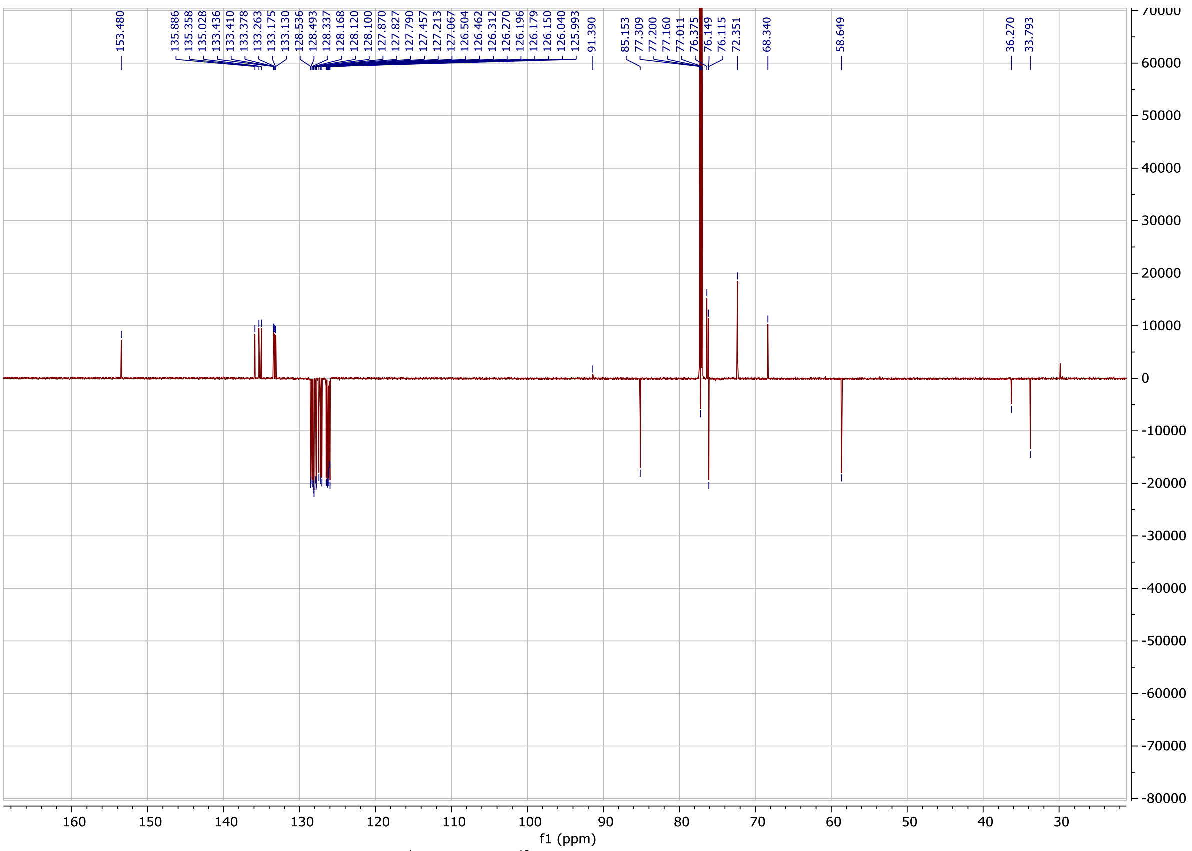

Figure S4:^{1}H-NMR and ^{13}C-NMR spectra of **24** in $CDCl_3$

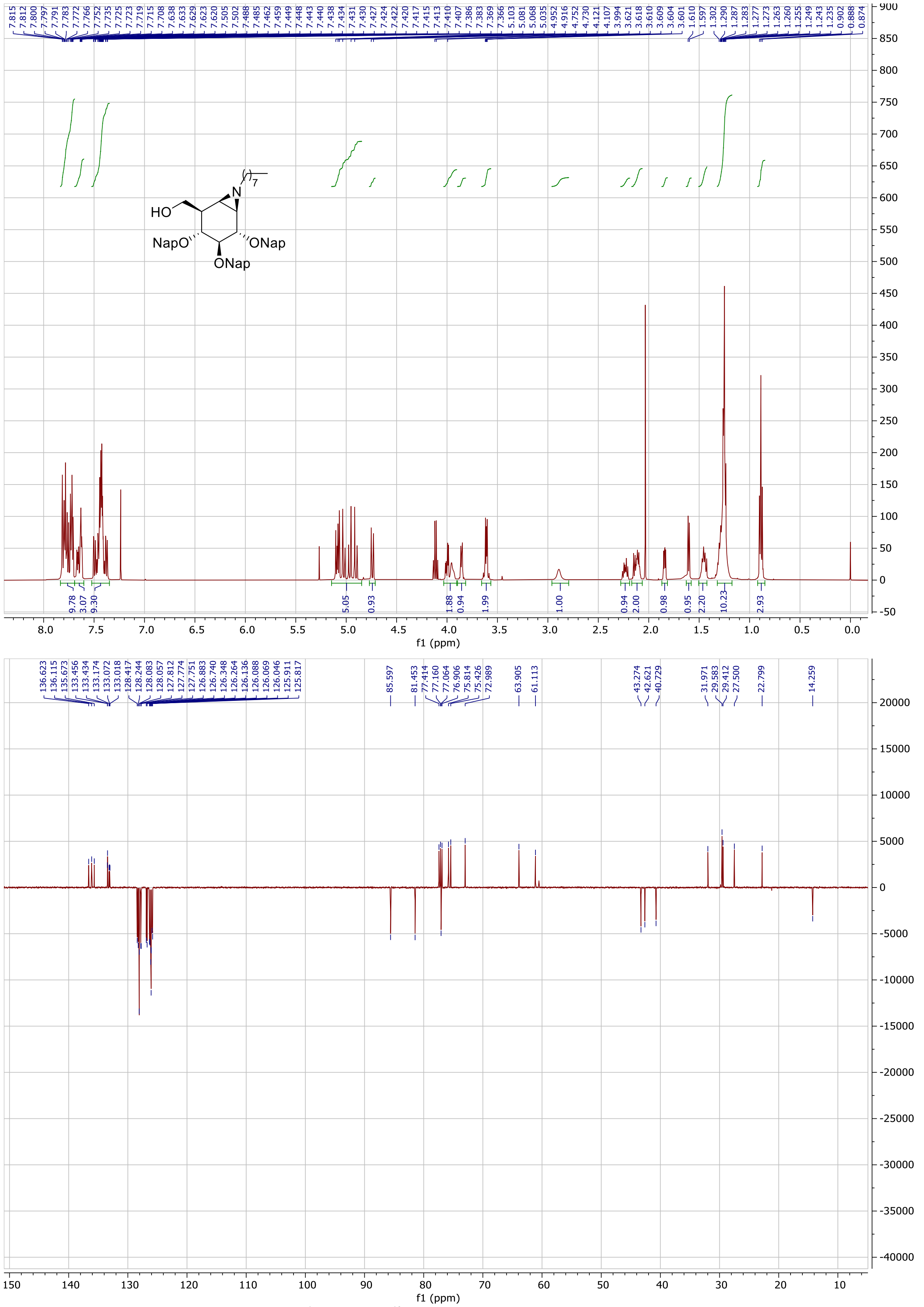

Figure S5: ^{1}H-NMR and ^{13}C-NMR spectra of **25** in $CDCl_3$

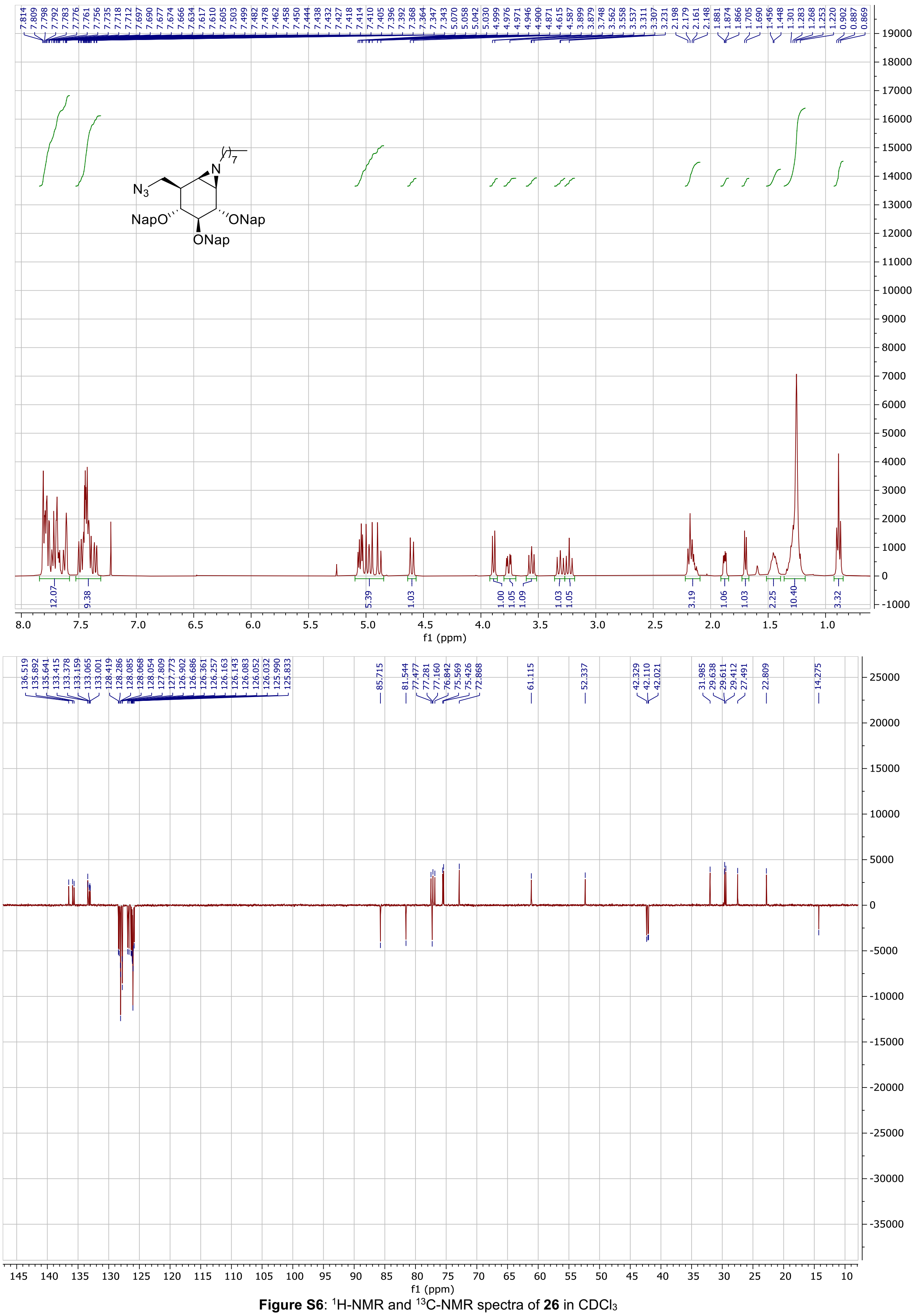

Figure S6: ^{1}H-NMR and ^{13}C-NMR spectra of **26** in $CDCl_3$

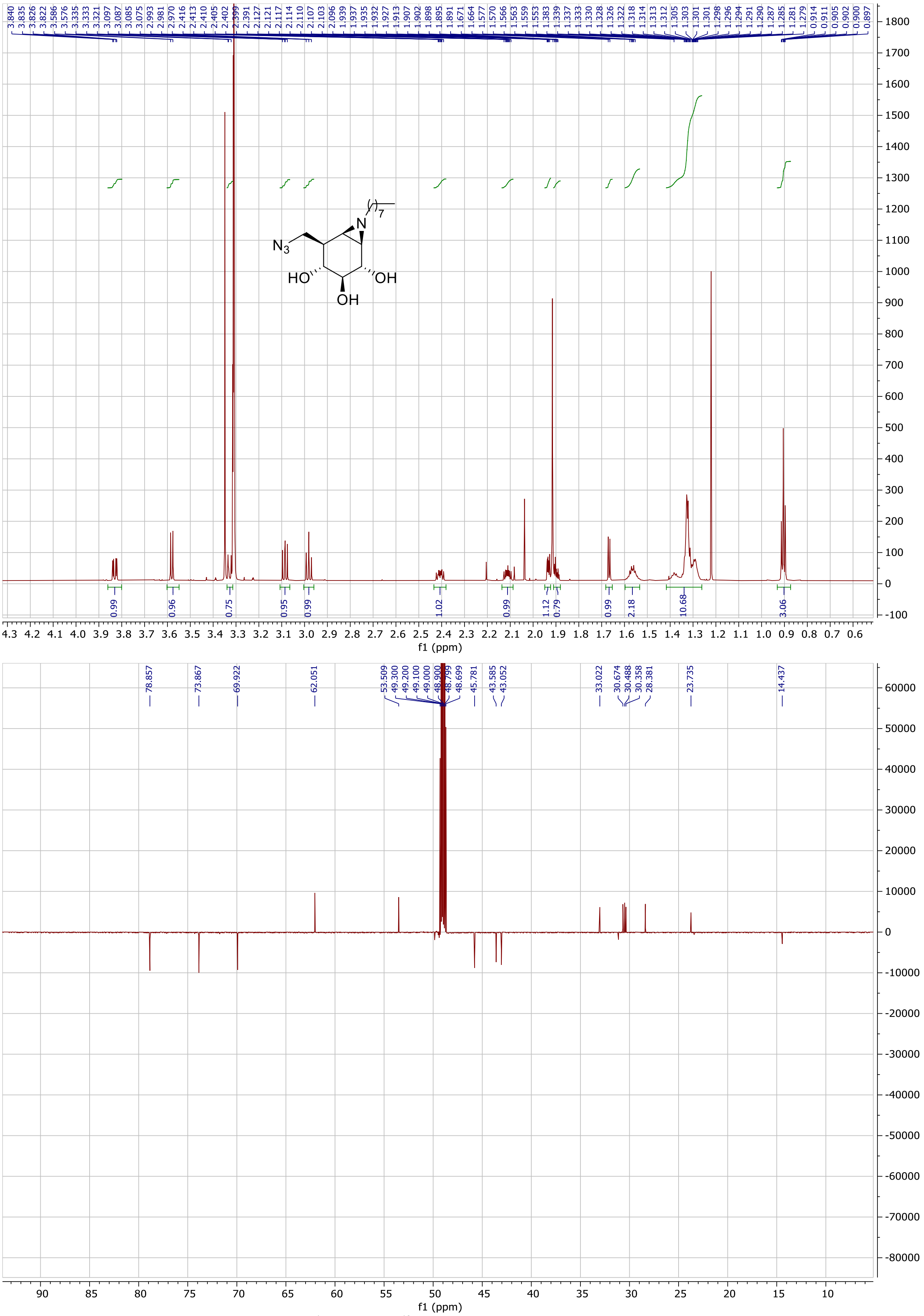

Figure S7: ^{1}H-NMR and ^{13}C-NMR spectra of **27** in MeOD

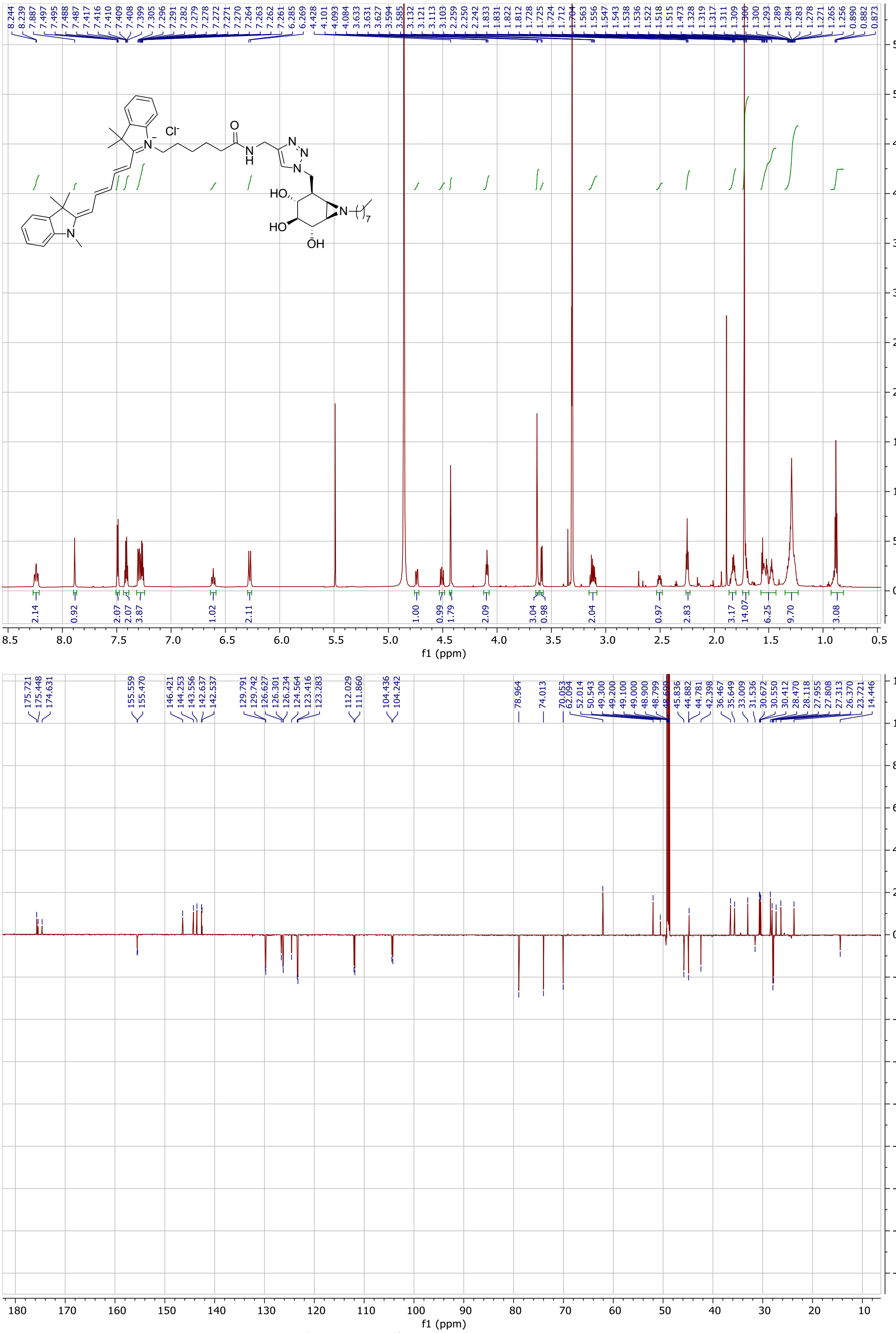

Figure S8: ^{1}H-NMR and ^{13}C-NMR spectra of **ABP 6** in MeOD

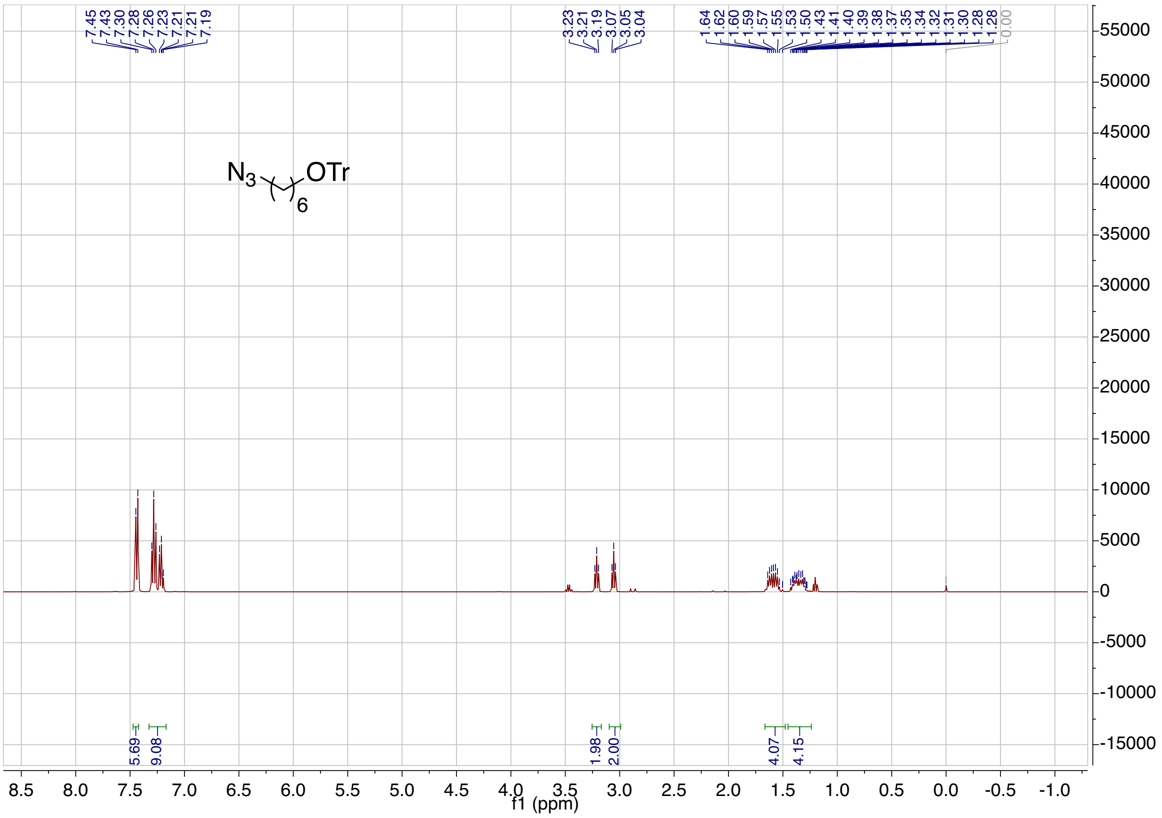

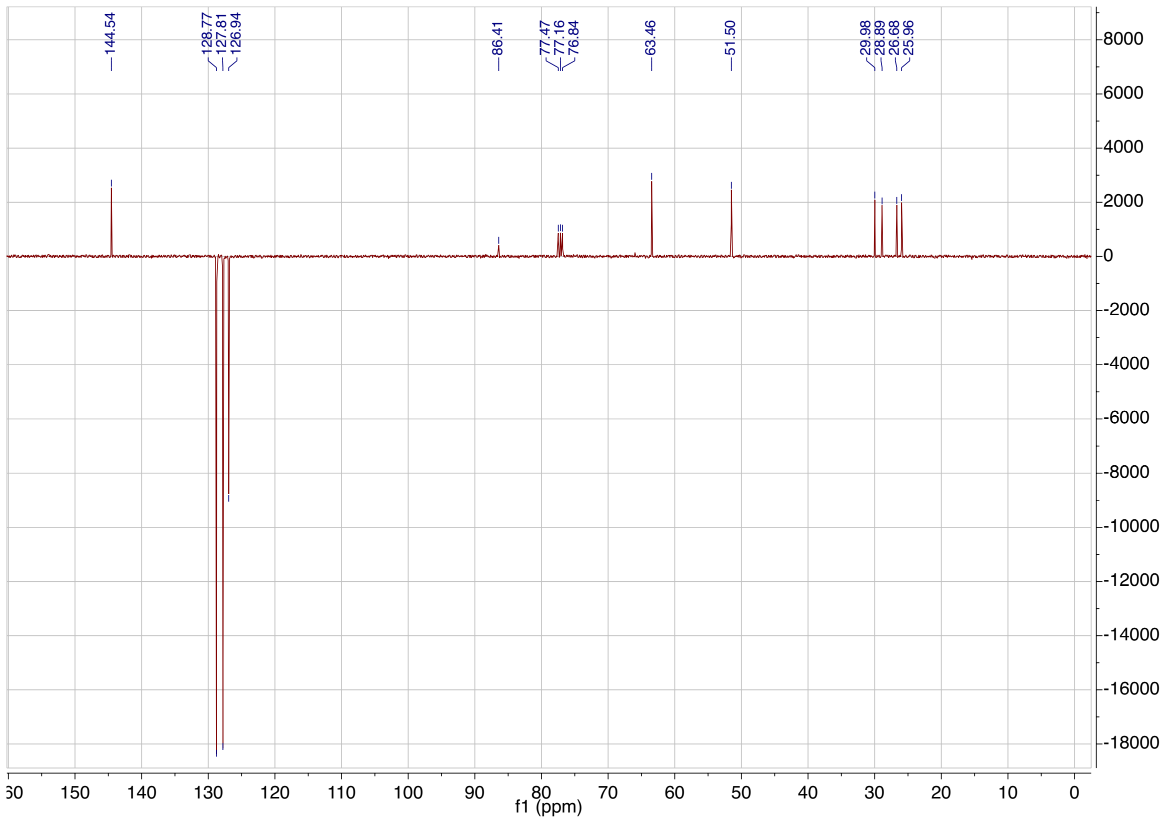

Figure S9: ^{1}H-NMR and ^{13}C-NMR spectra of **12** in $CDCl_3$

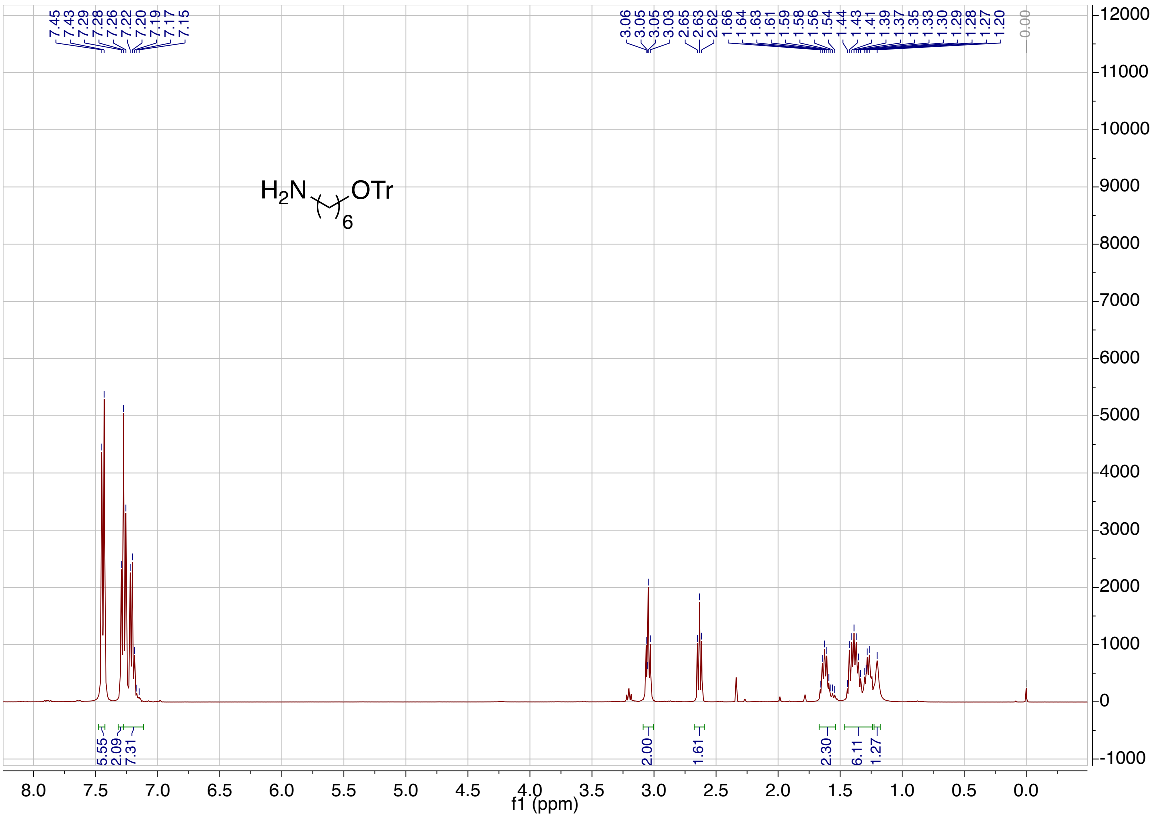

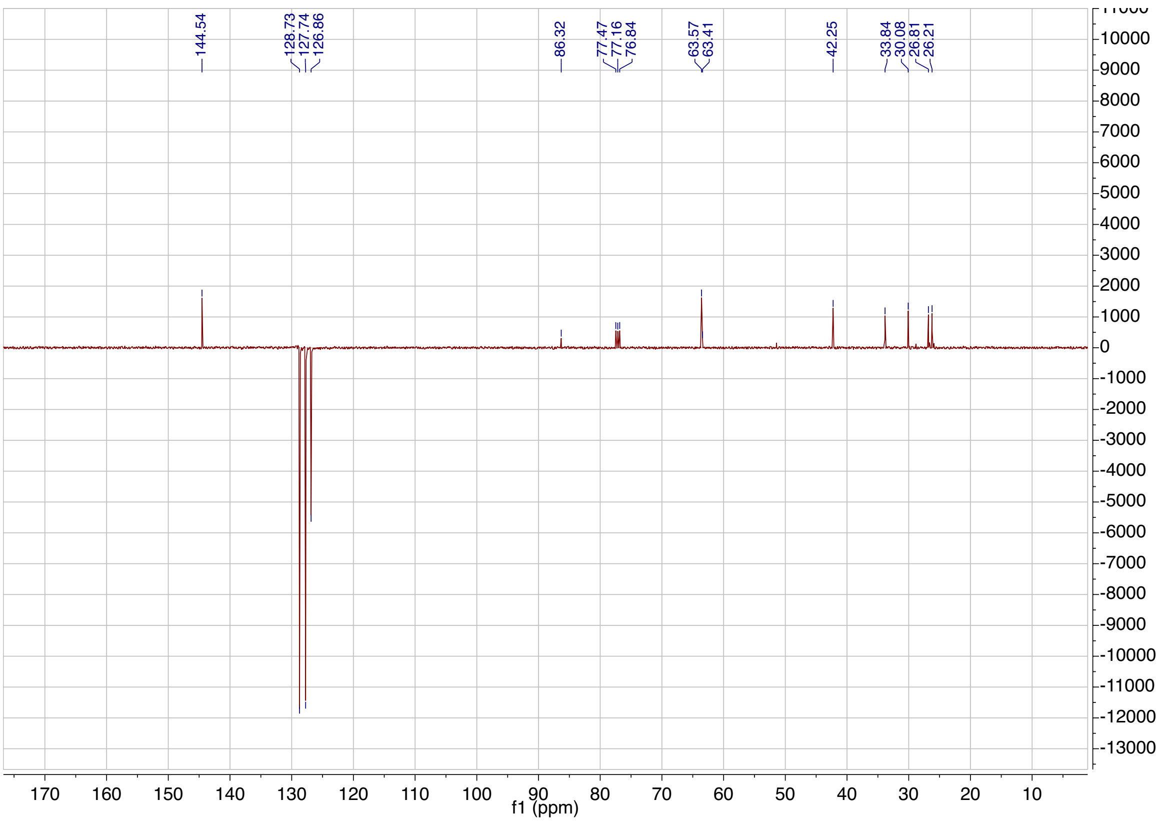

Figure S10: ^{1}H-NMR and ^{13}C-NMR spectra of **13** in $CDCl_3$

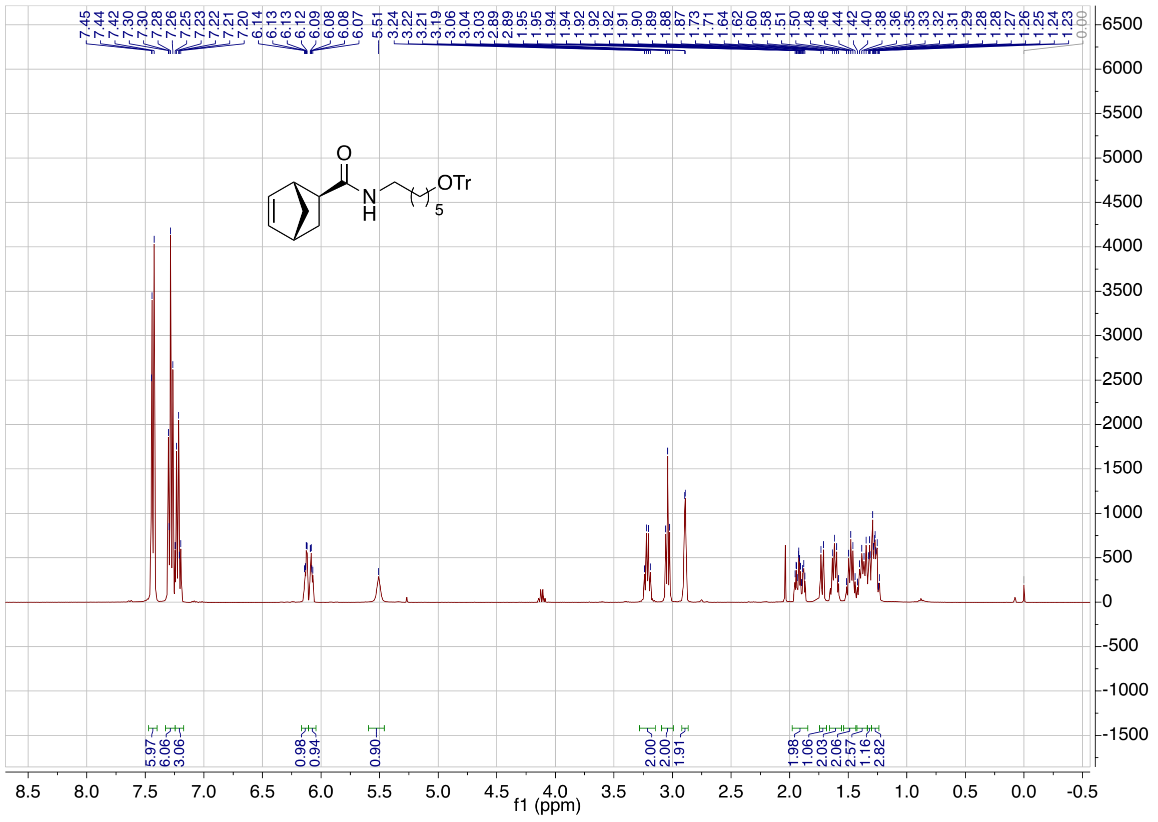

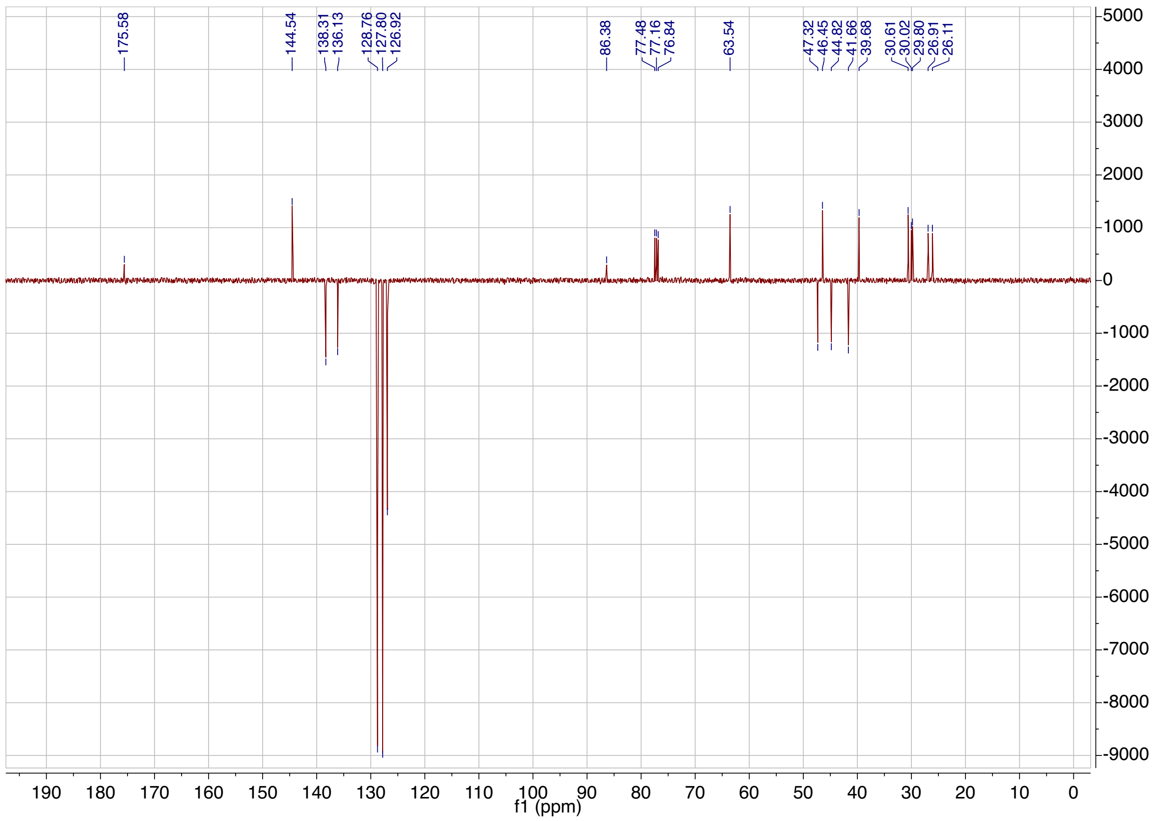

Figure S11: ^{1}H-NMR and ^{13}C-NMR spectra of **15** in $CDCl_3$

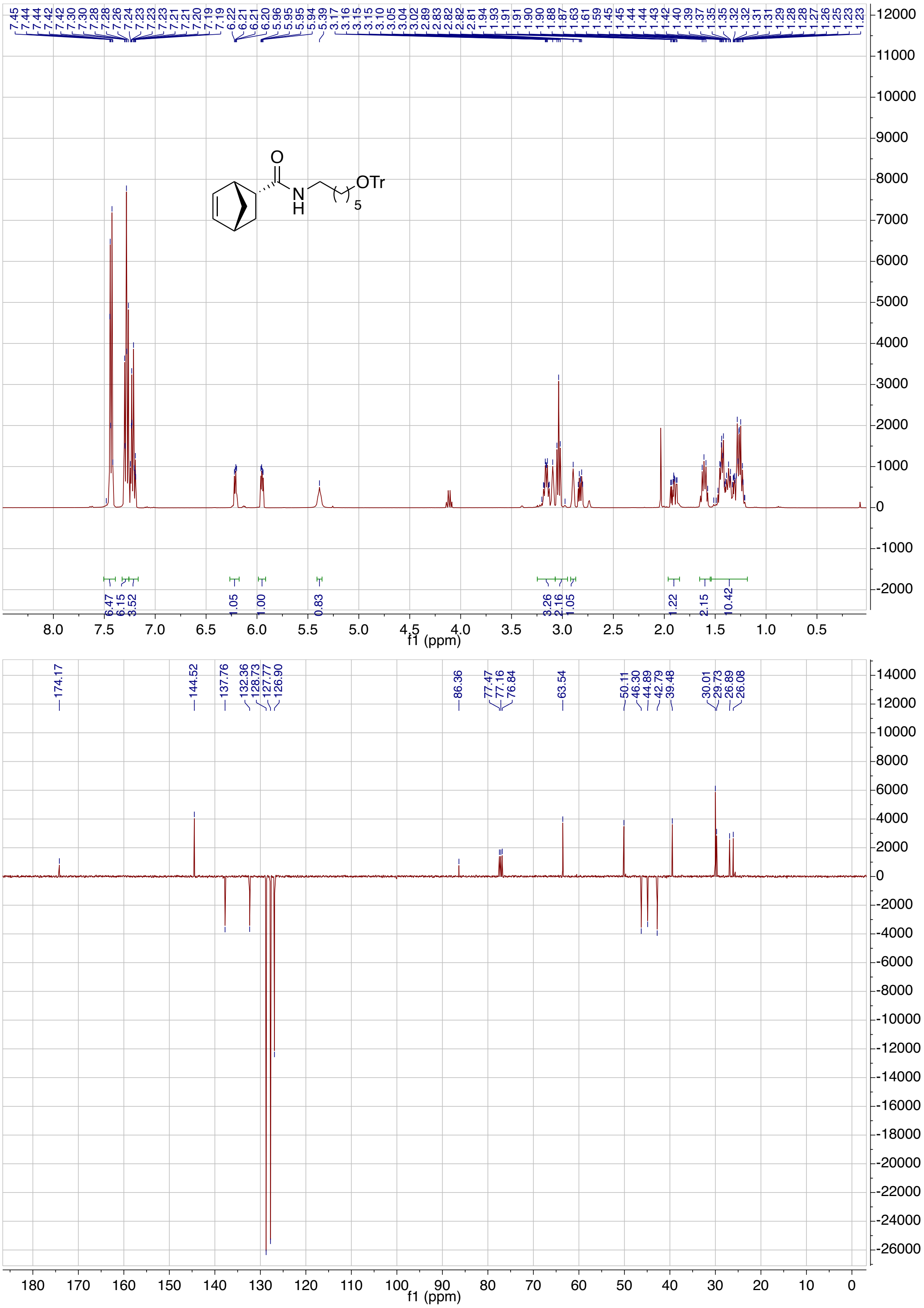

Figure S12: ^{1}H-NMR and ^{13}C-NMR spectra of **16** in $CDCl_3$

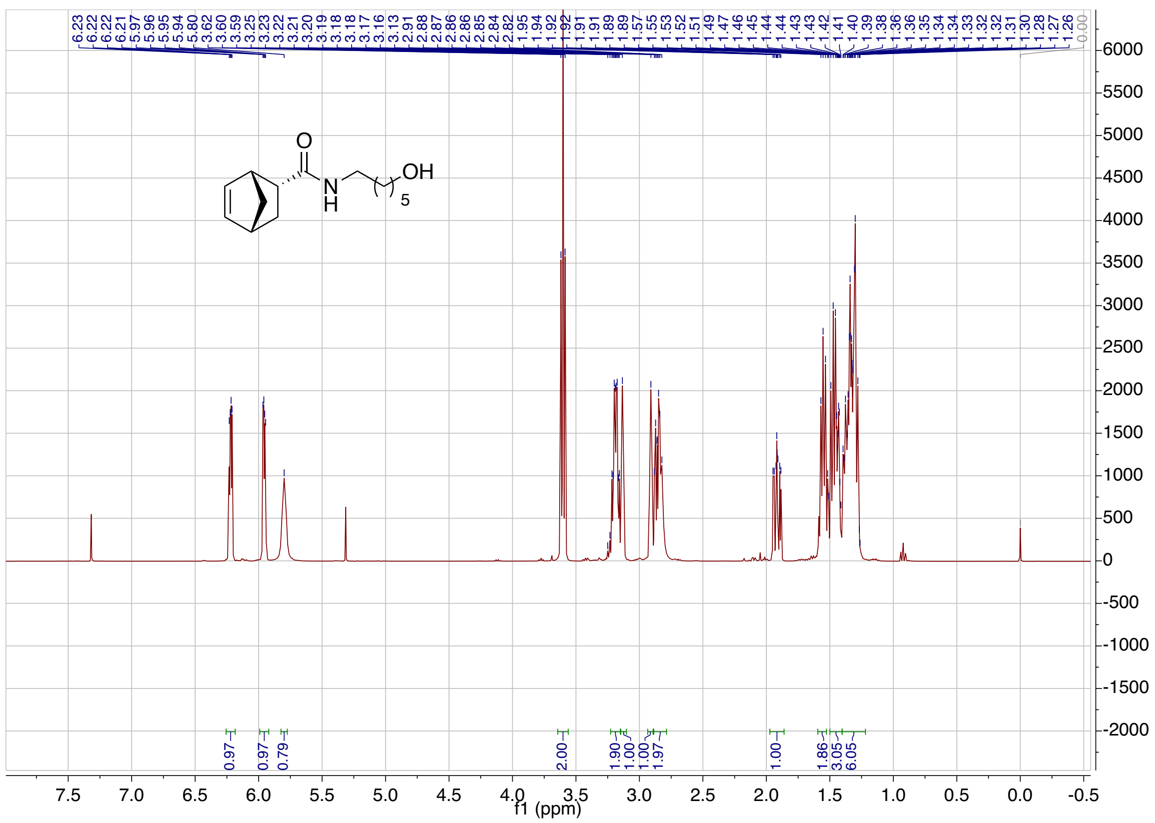

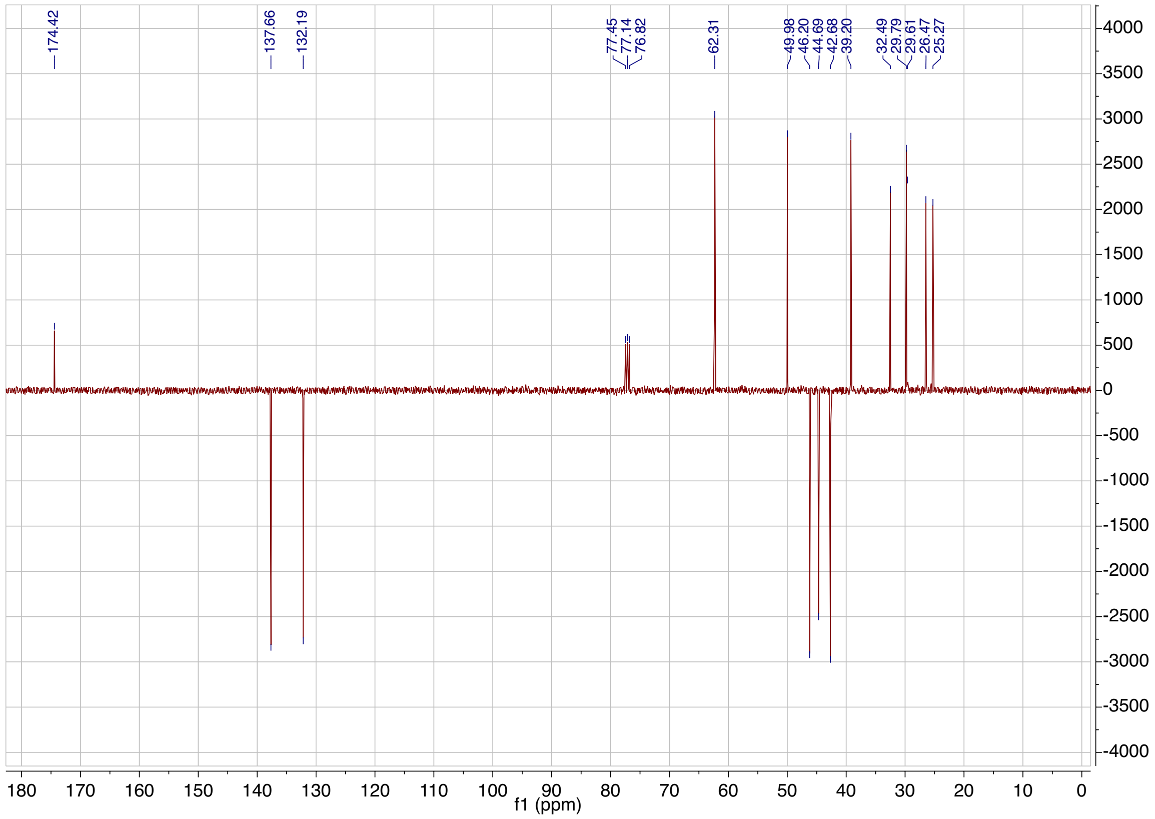

Figure S13:^{1}H-NMR and ^{13}C-NMR spectra of **17** in $CDCl_3$

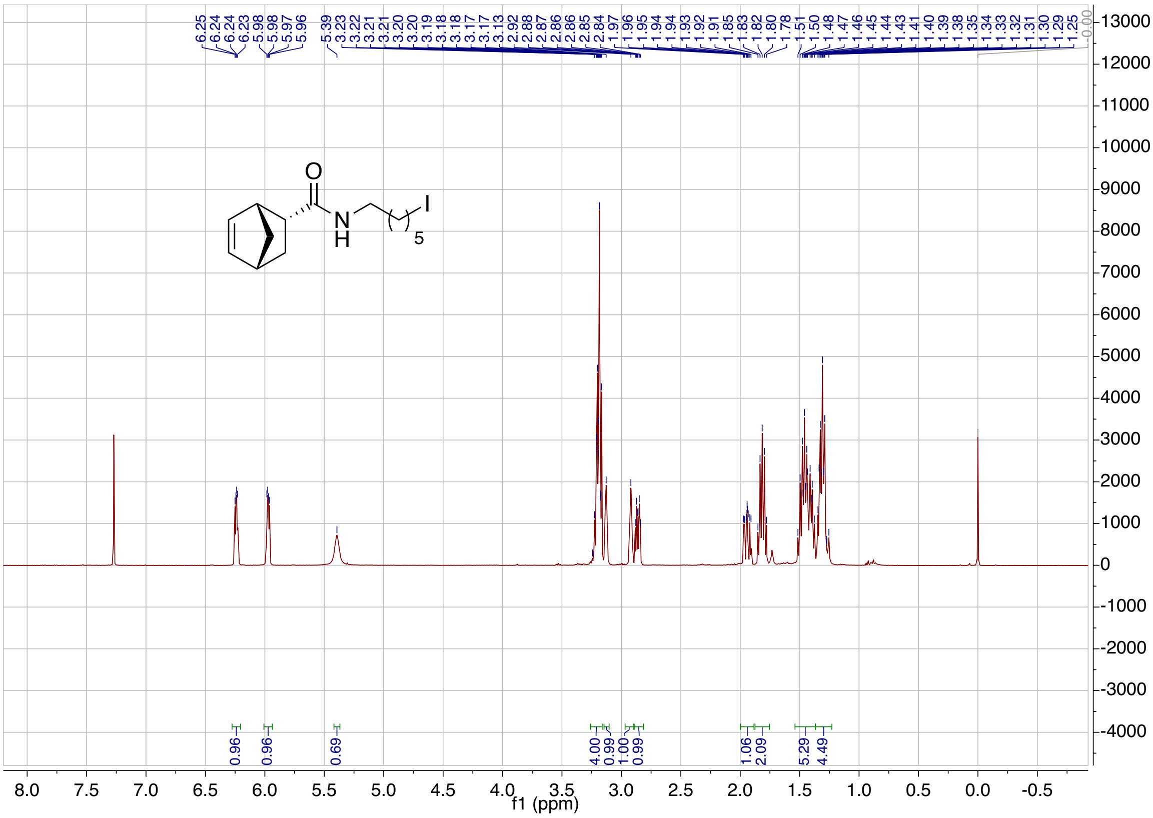

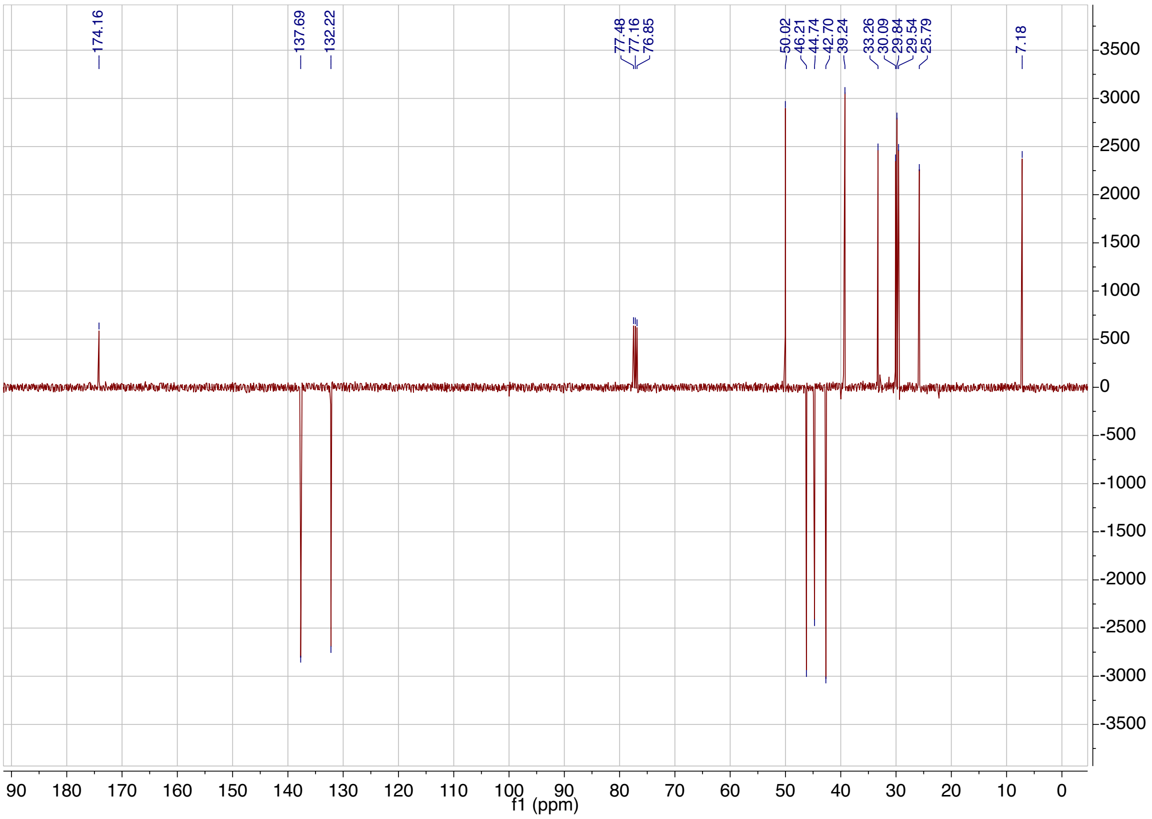

Figure S14: ^{1}H-NMR and ^{13}C-NMR spectra of **18** in $CDCl_3$

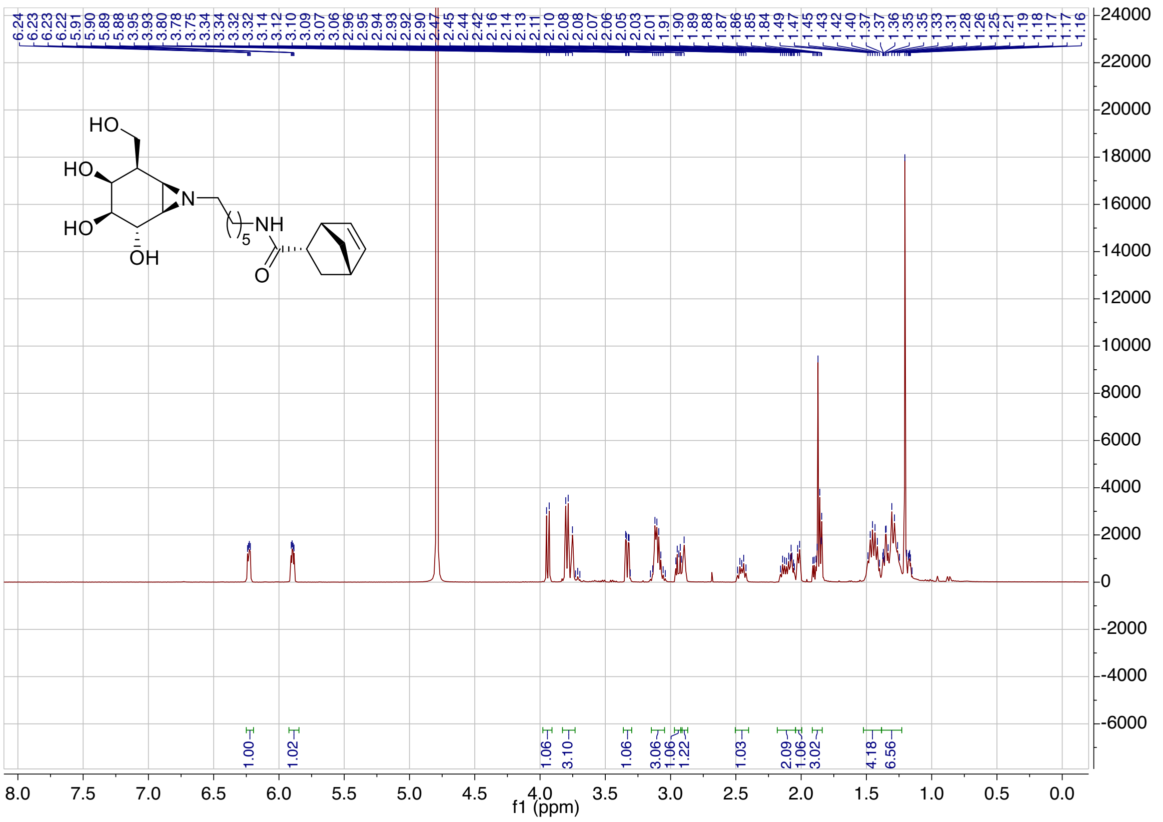

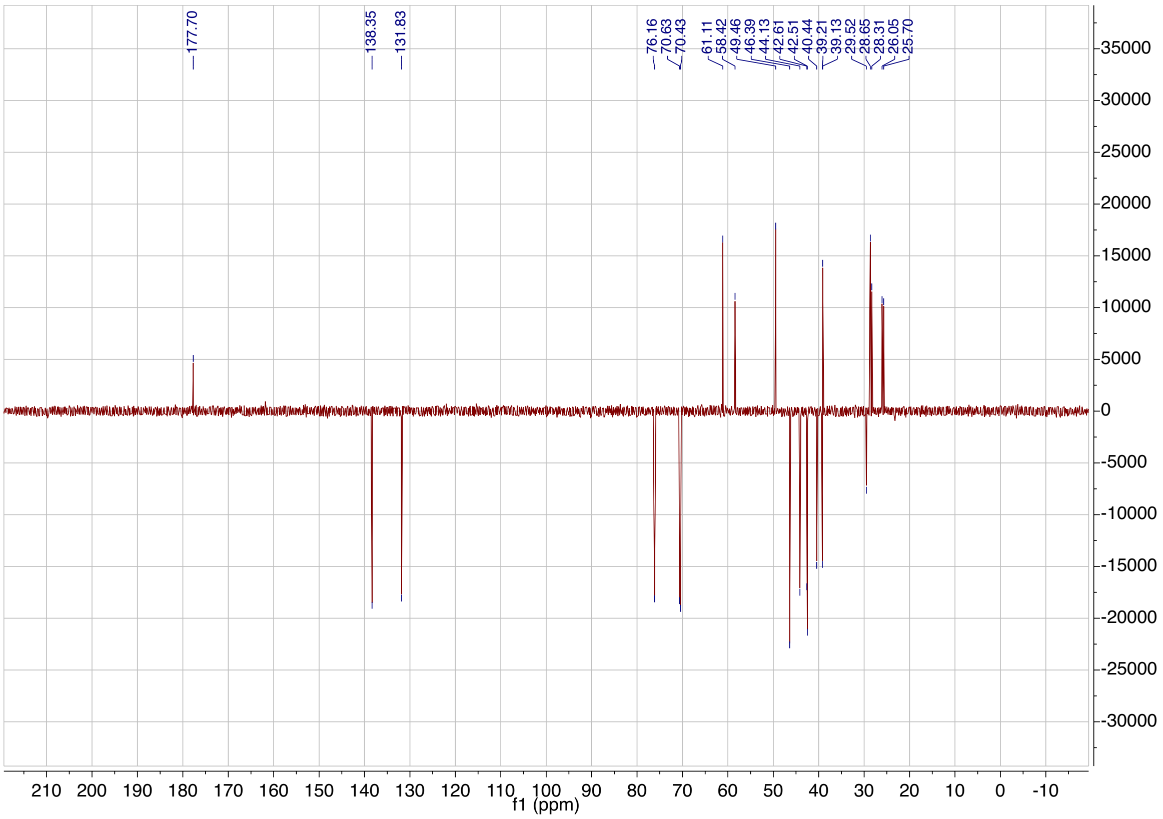

Figure S15: ^{1}H-NMR and ^{13}C-NMR spectra of **19** in D_2O

References

[1] R. Alibés, D. R. Bundle, *J. Org. Chem.* **1998**, *63*, 6288–6301.

[2] C. E. Yeom, Y. J. Kim, S. Y. Lee, Y. J. Shin, B. M. Kim, *Tetrahedron* **2005**, *61*, 12227–12237.

[3] P. A. Wallace, D. E. Minnikin, K. McCrudden, A. Pizzarello, *Chem. Phys. Lipids* **1994**, *71*, 145–162.

[4] L. I. Willems, N. Li, B. I. Florea, M. Ruben, G. A. van der Marel, H. S. Overkleeft, *Angew. Chemie Int. Ed.* **2012**, *51*, 4431–4434.

[5] D. Lahav, B. Liu, R. J. B. H. N. Van Den Berg, A. M. C. H. Van Den Nieuwendijk, T. Wennekes, A. T. Ghisaidoobe, I. Breen, M. J. Ferraz, C. L. Kuo, L. Wu, P. P. Geurink, H. Ovaa, G. A. Van Der Marel, M. Van Der Stelt, R. G. Boot, G. J. Davies, J. M. F. G. Aerts, H. S. Overkleeft, *J. Am. Chem. Soc.* **2017**, *139*, 14192–14197.

[6] M. Artola, C.-L. Kuo, L. T. Lelieveld, R. J. Rowland, G. A. van der Marel, J. D. C. Codée, R. G. Boot, G. J. Davies, J. M. F. G. Aerts, H. S. Overkleeft, *J. Am. Chem. Soc.* **2019**, *141*, 4214–4218.

[7] P. K. Smith, R. I. Krohn, G. T. Hermanson, A. K. Mallia, F. H. Gartner, M. D. Provenzano, E. K. Fujimoto, N. M. Goeke, B. J. Olson, D. C. Klenk, *Anal. Biochem.* **1985**, *150*, 76–85.
